# Decoding serotonin: the molecular symphony behind depression

**DOI:** 10.3389/fncel.2025.1572462

**Published:** 2025-04-24

**Authors:** Yue Shu, Lei Tian, Xing Wang, Tinyang Meng, Shouyang Yu, Yulan Li

**Affiliations:** ^1^The First School of Clinical Medicine, Lanzhou University, Lanzhou, China; ^2^Key Laboratory of Brain Science, Key Laboratory of Anesthesia and Organ Protection of Ministry of Education (In Cultivation), Zunyi Medical University, Zunyi, China

**Keywords:** depression, serotonin, serotonin transporter, tryptophan, tryptophan hydroxylase, vesicular monoamine transporter

## Abstract

The serotonin (5-hydroxytryptamine) system represents a crucial neurotransmitter network that regulates mood, behavior, and cognitive functions, playing a significant role in the pathogenesis and progression of depression. Although this perspective faces significant challenges, the serotonin system continues to exert substantial modulatory effects on specific aspects of psychological functioning and actively contributes to multiple pathological processes in depression development. Therefore, this review systematically integrates interdisciplinary research advances regarding the relationship between the 5-hydroxytryptamine (5-HT) system and depression. By focusing on core biological processes including serotonin biosynthesis and metabolism, SERT gene regulatory networks, and protein molecular modifications, it aims to elucidate how 5-HT system dysregulation contributes to the development of depression, while providing novel research perspectives and therapeutic targets for innovative antidepressant drug development.

## Graphical abstract

The association between the serotonin system and depression. AADC, aromatic amino acid decarboxylase; G, glycosyl; IDO, indoleamine 2,3-dioxygenase; KYN, kynurenine; P, phosphate; Pal, palmitoyl; Ub, ubiquitin; SERT, serotonin transporter; SIP, SERT interacting protein; SNP, single nucleotide polymorphism; TDO, tryptophan 2,3-dioxygenase; Tph, tryptophan hydroxylase.
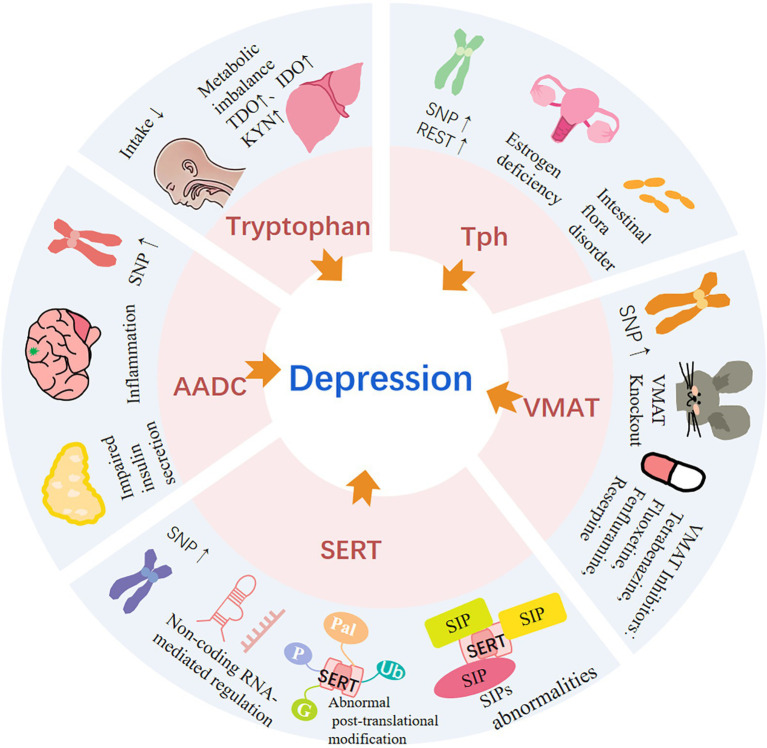

## 1 The monoamine hypothesis of depression

**Figure 1:**
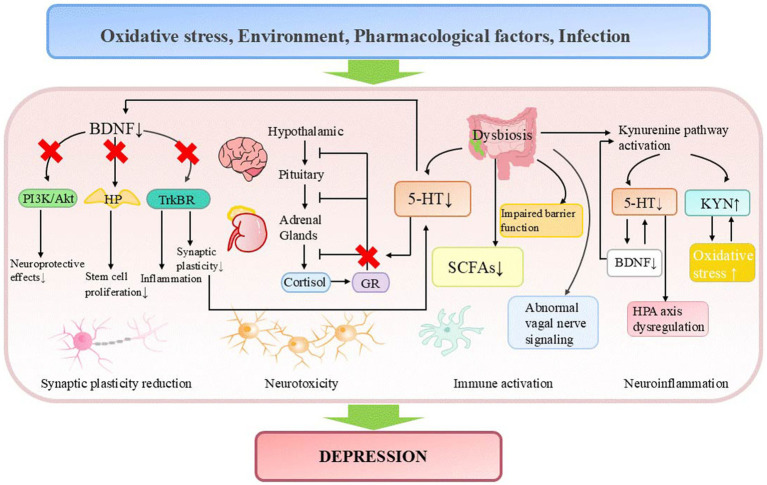
Pathogenesis of depression. 5-HT, serotonin; BDNF, brain-derived neurotrophic factor; GR, glucocorticoid receptor; HP, hippocampus; HPA axis, hypothalamic-pituitary-adrenal axis; KYN, kynurenine; SCFAs, short-chain fatty acids.

Depressive disorders (also known as depression) are characterized by depressive mood (e.g., sad, irritable, empty) or loss of pleasure accompanied by other cognitive, behavioral, or neurovegetative symptoms that significantly affect the individual’s ability to function. According to statistics, approximately 280 million people worldwide suffer from depression, accounting for 3.8% of the global population (Wróbel et al., 2024). Currently, several widely recognized hypotheses aim to explain the pathogenesis of depression. These include the monoamine hypothesis (Li, 2024), the neuroplasticity hypothesis (Chen et al., 2024), the inflammation hypothesis (Lemogne et al., 2024), the hypothalamic-pituitary-adrenal (HPA) axis dysfunction hypothesis (He et al., 2024), the glutamate-GABA imbalance hypothesis (Bi et al., 2024), and the gene-environment interaction hypothesis (Ejiohuo et al., 2025) (Figure 1). Among these, the monoamine hypothesis is one of the earliest accepted theories. In the 1950s, doctors accidentally discovered that isoniazid (Isoniazid) and iproniazid (Iproniazid), which were used to treat tuberculosis, could improve the depressive symptoms of patients (Loomer et al., 1957). In contrast, the antihypertensive drug reserpine (Reserpine) led to severe depressive symptoms in some patients (Freis, 1954). Coincidentally, these drugs all altered monoamine signaling. It was only then that people first connected changes in monoamines to mood regulation. Later, Schildkraut (1965) summarized the research data at the time and proposed that norepinephrine deficiency might lead to the development of depression. Coppen (1967) also systematically explored the role of serotonin (5-HT) in mood regulation and suggested the possibility of treating depression by modulating 5-HT levels. They believed that dysfunction or dysregulation of the monoamine system could be one of the pathological bases of depression, which ultimately led to the formation of the monoamine hypothesis. As researchers continue to explore the 5-HT system, it has gradually become recognized as one of the most important targets for treating depression, anxiety, panic, mood disorders, and other mental illnesses (Hung L. Y. et al., 2024). Since various antidepressants have highly similar effects on 5-HT levels (Witt et al., 2023), 5-HT is also considered a common link connecting different types of antidepressants (Witt et al., 2023), making it a key neurotransmitter related to the pathophysiology of depression.

In recent decades, the monoamine hypothesis of depression has been increasingly challenged due to several limitations observed in antidepressant drugs developed under this framework. These limitations encompass delayed therapeutic onset, treatment resistance in certain patient populations, and the theory’s inability to account for the underlying causes of neurotransmitter dysregulation. After reviewing the data in the field of 5-HT research, Moncrieff et al. (2023) concluded that there is no convincing evidence to suggest that depression is related to or caused by decreased 5-HT levels or activity, and raised questions about the high usage rates of antidepressant drugs. Shortly thereafter, this study encountered substantial opposition from the scientific community, primarily attributed to deficiencies in its assessment methodology, inaccurate interpretation of clinical pharmacological significance, and erroneous analysis of molecular imaging data related to serotonin transporter binding (Jauhar et al., 2023; Royal College of Psychiatrists, 2019). Ongoing research has identified multiple pathogenic factors in depression, including impaired neuroplasticity, inflammatory dysregulation, neuroimmune dysfunction, disrupted BDNF signaling, and HPA axis abnormalities (Chen et al., 2024; Lemogne et al., 2024; He et al., 2024; Köhler et al., 2017; Duman and Aghajanian, 2012; Castrén and Rantamäki, 2010). For instance, depressed patients frequently exhibit volume reductions in the hippocampus and prefrontal cortex, while antidepressant treatment upregulates neuronal plasticity-related molecules and enhances neurogenesis and synaptic density in these regions (Park et al., 2023). Clinical studies demonstrate elevated peripheral levels of pro-inflammatory cytokines (IL-6, TNF-α, IL-10) in major depressive disorder (MDD, which is a clinically diagnosed condition, while depression typically refers to depressive symptoms without a clinical diagnosis) (Köhler et al., 2017). Chronic unpredictable mild stress (CUMS) induces cortisol elevation and depressive-like behaviors in mice (Xu et al., 2025). Serotonin deficiency may interact with these pathological mechanisms, collectively driving disease progression. Specifically, monoamines participate in neuroimmune regulation by modulating microglial activity and cytokine release (Miller and Raison, 2016), while also maintaining neuronal development, synaptic plasticity, BDNF signaling, and HPA axis homeostasis through regulation of glucocorticoid receptor (GR) expression and function (Bagdy and Makara, 1994; Pariante and Lightman, 2008). Chronic monoamine deficiency may impair synaptic structure and function by suppressing BDNF signaling (Xu et al., 2000), compromising neuroprotective effects (Liu et al., 2021), and inhibiting hippocampal stem cell proliferation (Park et al., 2023). Additionally, it may disrupt HPA axis negative feedback regulation, exacerbating cortisol-induced neurotoxicity and neuronal damage. Together, these effects drive the pathogenesis of depression (Farooqi et al., 2018) (Figure 1).

While the chemical imbalance hypothesis might be considered reductionistic in accounting for the cerebral alterations associated with depression, it remains indispensable across various etiological theories of the disorder. This conceptual model contributes to our understanding of specific psychological processes and offers essential theoretical support and clinical direction for depression research and treatment. Consequently, this review systematically integrates cross-disciplinary research progress regarding the association between the 5-HT system and depressive disorders. By investigating key processes including serotonin biosynthesis and metabolism, serotonin transporter (SERT) gene regulation, and protein post-translational modifications, this study aims to elucidate how 5-HT system dysregulation contributes to the development of depression. These findings will advance the development of early diagnostic approaches, improve mechanistic understanding of the disease, and facilitate targeted therapeutic interventions.

## 2 Discovery and composition of the serotonin system

Serotonin, also known as 5-HT, is one of the oldest neurotransmitters. Its discovery can be traced back to the 1930s. In 1937, the Italian scientist Vialli and Erspamer (1937) discovered a chemical substance found in the enterochromaffin cells of the intestine, which had a strong smooth muscle contraction effect. He named it enteramine (meaning “intestinal amine”). Ten years later, Maurice isolated a substance from bovine serum that could induce vasoconstriction and named it serotonin (Rapport et al., 1948). In 1952, Erspamer and Asero (1952) successfully isolated and purified enteramine and identified its chemical structure as 5-HT. Further research confirmed that “enteramine” and “serotonin” were the same substance. Subsequently, scientists discovered the presence of 5-HT in both the central nervous system and peripheral tissues (Nair, 1965; Anton and Gennaro, 1965). In 1967 and 1972, the two catalytic enzymes necessary for 5-HT synthesis—tryptophan hydroxylase (Tph) (Lovenberg et al., 1967) and aromatic L-amino acid decarboxylase (AADC) (Christenson et al., 1970)—were discovered. In 1957, the 5-hydroxytryptamine receptor (5-HTR) was identified for the first time. Gaddum and Picarelli (1957) proposed two distinct mechanisms of 5-HT action on smooth muscle and attributed them to two different types of receptors, referred to as “D” and “M” types. It was not until 1979 that Peroutka and Snyder (1979), through radioligand binding experiments, first identified the existence of 5-HTR1 and 5-HTR2. He proposed that 5-HTR1 is an inhibitory receptor that reduces cyclic adenosine monophosphate (cAMP) levels, while 5-HTR2 is an excitatory receptor that activates phospholipase C. A few years later, the 5-HTR3 was discovered and identified as the only ligand-gated ion channel receptor, differing from the traditional G protein-coupled receptors (GPCRs) (Barnes et al., 1989). In the 1990s, molecular cloning techniques confirmed the existence of new subtypes such as 5-HTR4, 5-HTR5, 5-HTR6, and 5-HTR7 (Hoyer et al., 1994). To date, seven receptor families comprising 14 subtypes of 5-HTR have been identified in mammals (Brunetti et al., 2024).

## 3 Synthesis and metabolism of serotonin

5-HT is distributed in both the central and peripheral tissues. Since it cannot cross the blood–brain barrier, two relatively independent systems are required for its synthesis.

**Figure 2:**
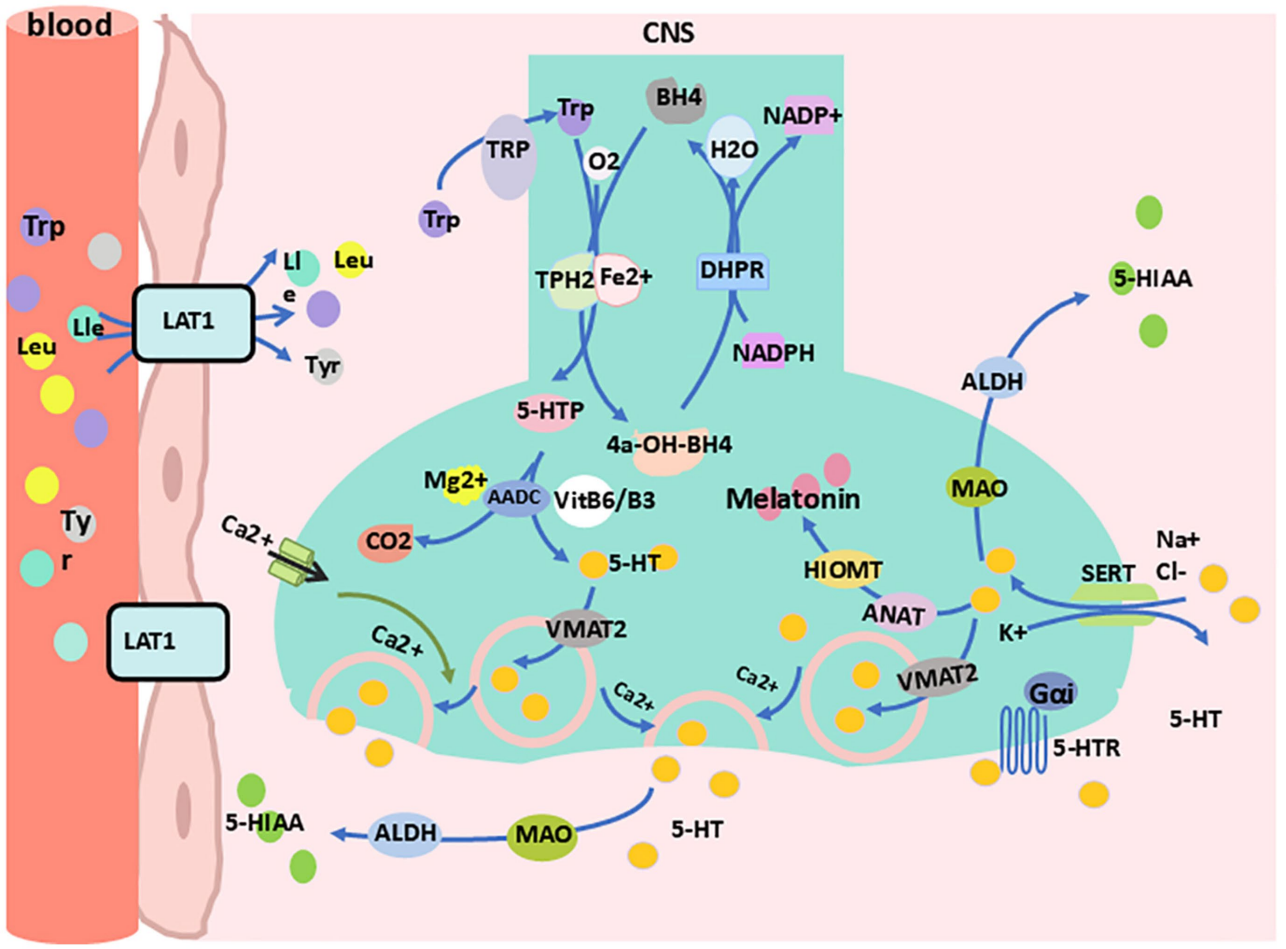
Synthesis and metabolism of 5-HT in the central nervous system. 4a-OH-BH4, 4a-hydroxy-tetrahydrobiopterin; 5-HIA, 5-hydroxyindoleacetic acid; 5-HTp, 5-hydro-xytryptophan; 5-HTR, 5-HT receptor; AADC, aromatic L-amino acid decarboxylase; ALDH, aldehyde dehydrogenase; ANAT, arylalkylamine N-acetyltransferase; BH4, tetrahydrobiopterin; DHPR, dihydropteridine reductase; Gαi, G proteins alpha inhibitory subunit; HIOMT, hydroxyindole-O-methyltransferase; LAT1, L-type amino acid transporter 1; Lle, lsoleucine; Leu, leucine; MAO, monoamine oxidase; NADP^+^, nicotinamide adenine dinucleotide phosphate; SERT, serotonin transporter; Tp-h2, tryptophan hydroxylase 2; TRP, transient receptor potential; Trp, tryptophan; Tyr, tyrosine; VitB6/B3, vitamin B6/vitamin B3; VMAT2, vesicular monoamine transporter 2.

In the central nervous system, 5-HT is primarily synthesized in the raphe nuclei. Dietary tryptophan competes with leucine, isoleucine, and other neutral amino acids for neutral amino acid transporters to cross the blood-brain barrier and enter the central nervous system. Once inside, tryptophan is taken up by 5-HT nerve terminals in the raphe nuclei (Ahmed, 2024; Steinbusch et al., 2021). The absorbed tryptophan is then converted into 5-HT through the actions of Tph2 and AADC (Specker et al., 2023; Baribeau et al., 2024). Subsequently, a portion of the synthesized 5-HT is rapidly transported into vesicles by vesicular monoamine transporters (Vmat) and stored (Hung L. Y. et al., 2024). The remaining intracellular free 5-HT is metabolized by monoamine oxidase (MAO) and aldehyde dehydrogenase (ALDH) into 5-hydroxyindoleacetic acid (5-HIAA) (Best et al., 2010). When neurons are mechanically or chemically stimulated, 5-HT stored in vesicles is rapidly released into the synaptic cleft. In the synaptic cleft, part of the free 5-HT binds to downstream receptors to exert its effects, part is reabsorbed into the cell through the presynaptic SERT, and the rest is further metabolized extracellularly into 5-HIAA (Best et al., 2010) (Figure 2). Meanwhile, presynaptic 5-HT autoreceptors regulate the synthesis and release of intracellular 5-HT through negative feedback by sensing changes in extracellular 5-HT levels (Ansari et al., 2024). These raphe nuclei 5-HT neurons project extensively throughout the brain, participating in functions such as mood regulation, sleep-wake control, appetite regulation, pain perception, and motor coordination (Mao et al., 2024; Wang et al., 2024; Tahiri et al., 2024; Hao et al., 2023). Notably, 5-HT within the pineal gland cells is further converted into melatonin under enzymatic action, contributing to sleep-wake regulation (Xia et al., 2024).

In peripheral tissues, 5-HT is primarily synthesized by enteric nerve cells, with a small portion synthesized by enterochromaffin-like cells, mast cells, and other cells (Mao et al., 2024; Roumier et al., 2019; Mawe et al., 2023). Dietary tryptophan enters the intestinal lumen, is absorbed into cells via the amino acid transport system of enterochromaffin cells, and is then converted into 5-HT through the actions of Tph1 and AADC (Kannen et al., 2020). Enterochromaffin cells release 5-HT into the lamina propria, where part of the 5-HT is reabsorbed by nearby enterocytes and goblet cells, another portion binds to the receptors of the enterochromaffin cells themselves, and a third interacts with sensory nerve terminals or immune cells. The remaining 5-HT is absorbed by platelets into the bloodstream (Kannen et al., 2020). After 5-HT binds to receptors on sensory nerve terminals (mainly 5-HTR3 and 5-HTR1), it transmits signals to the central nervous system (spinal nerves) and the prevertebral sympathetic ganglia (vagal afferents) for various processing (Wood, 2007). When 5-HT interacts with immune cells, it induces changes in downstream inflammatory factors (Roumier et al., 2019; Kannen et al., 2020). The remaining 5-HT that enters the bloodstream is partly metabolized in a free form by liver enzymes (Mao et al., 2024) and partly stored in platelet cell granules. When tissue damage or acute inflammation occurs, platelets are activated and release 5-HT into the blood. On one hand, 5-HT promotes platelet aggregation and peripheral vasoconstriction, participating in hemostasis (Berger et al., 2009). On the other hand, 5-HT regulates immune responses by inducing lymphocyte proliferation, modulating cytokine release, and recruiting neutrophils to the injury site to combat infection (Mauler et al., 2016). Additionally, serotonin is involved in processes such as gastrointestinal regulation (Kannen et al., 2020), mammary gland development (Fayyaz et al., 2024), vascular tone regulation (Wabel et al., 2024), bone density (Bulut et al., 2024), and heart rate control (Sheng et al., 2024).

## 4 The 5-HT system and depression

### 4.1 5-HT

While not the sole driver, 5-HT deficiency and metabolic dysregulation contribute to certain aspects of depression, such as modulating emotional processing. This phenomenon was initially observed as early as the 1950s. Reserpine, by depleting the concentrations of monoamine neurotransmitters (primarily catecholamines and serotonin) in the brain, has been observed to induce significant depressive symptoms in some patients (Freis, 1954). This phenomenon provided support for the “monoamine hypothesis” of depression and marked the beginning of understanding the role of 5-HT in the disorder. Subsequently, both animal models and human studies have consistently demonstrated that serotonin deficiency often leads to depression-like behaviors. Saldanha et al. (2009) found that serum 5-HT levels in patients with depression were significantly lower than those in healthy individuals. Postmortem analysis of brain tissues from individuals with MDD, who exhibited suicidal tendencies revealed increased expression of 5-HTR1A in the dorsolateral nucleus, a phenomenon that may also be attributed to reduced 5-HT levels in this brain region (Stockmeier, 1997). Additionally, chronic stress has been shown to induce a significant reduction in 5-HT release in both plasma and multiple brain regions of experimental animals, accompanied by the emergence of depression-like behaviors (Ahmad et al., 2010; Yi et al., 2008). Furthermore, researchers have observed dysregulated levels of 5-HIAA, a major metabolite of 5-HT, in the cerebrospinal fluid and urine of patients with depression, which were significantly correlated with the severity of depressive symptoms (Asberg et al., 1976; Bhatia et al., 1978). This finding suggests that 5-HT metabolism may be impaired in individuals with depression. In subsequent studies, researchers further discovered that patients with MDD exhibited significantly elevated levels of MAO-A distribution throughout the brain (Meyer et al., 2006), while peripheral MAO activity was also increased (Georgotas et al., 1986; Fähndrich et al., 1982). Collectively, these findings suggest that elevated serotonin metabolism may be actively involved in the pathogenesis of depression.

The excessive activation of MAO-A, rapid degradation of 5-HT, and subsequent elevation of 5-HIAA levels may lead to a decline in serotonin levels, thereby impairing mood regulation and cognitive function and potentially triggering or exacerbating depressive symptoms. Consequently, targeting the modulation of 5-HT metabolism has emerged as a significant therapeutic strategy in the treatment of depression. First-generation antidepressants, such as phenelzine and brofaromine, function as MAO inhibitors. They increase the concentrations of monoamine neurotransmitters (including serotonin and norepinephrine) in the brain and reduce 5-HT degradation, often leading to significant improvement in depressive symptoms (Celada et al., 1992). Notably, phenelzine has regained widespread attention in the fields of neuroscience and psychopharmacology due to its additional neuroprotective and antioxidative stress properties (Baker et al., 2012; Harvey et al., 2017). Subsequently, researchers developed selective serotonin reuptake inhibitors (SSRIs), aiming to reduce serotonin metabolism and alleviate depression by blocking 5-HT reuptake. This approach has also demonstrated significant antidepressant efficacy (Talkowski et al., 2008). However, its therapeutic effects are often limited by the overactivation and hypersensitivity of 5-HTR1A, leading to a delayed improvement in mood during the initial stages of treatment (Sharp and Barnes, 2020). Although combining potent 5-HTR1A antagonists with SSRIs has shown potential to enhance antidepressant effects, this strategy requires further support from high-quality randomized controlled trials to confirm its applicability and safety. Additionally, the efficacy of SSRIs is influenced by the kynurenine pathway. Although SSRIs increase synaptic concentrations of 5-HT, excessive activation of the kynurenine pathway can divert tryptophan metabolism toward the production of kynurenine, thereby reducing the availability of tryptophan for 5-HT synthesis. This mechanism may compromise the therapeutic effects of SSRIs (Rampersaud et al., 2025). The activation of the kynurenine pathway is often associated with immune system activation, which may further exacerbate inflammatory responses in patients with depression, thereby diminishing the antidepressant effects of SSRIs (Wichers et al., 2005). Consequently, therapeutic strategies targeting the kynurenine pathway have emerged as a promising new direction for the treatment of depression, offering potential clinical value.

### 4.2 Tryptophan

Tryptophan, as the precursor for 5-HT synthesis, plays a critical role in determining the rate of 5-HT production based on its concentration and bioavailability (Correia and Vale, 2022). Abnormal levels of tryptophan in the body are considered a significant factor in the development of mood disorders. Since the human body cannot synthesize tryptophan, its levels are primarily influenced by dietary intake (Correia and Vale, 2022; Poeggele et al., 2022). Researchers have found that elderly patients with mild to moderate depression have lower tryptophan intake from food (Chojnacki et al., 2020). Healthy individuals who consume low levels of tryptophan are also more susceptible to depression (Lindseth et al., 2015). However, after appropriately increasing tryptophan intake, their emotional disorders improve (Lindseth et al., 2015; Chojnacki et al., 2023) and the incidence of depression and pancreatic cancer decreases (Hung N. L. et al., 2024; Suga et al., 2018). Therefore, regulating tryptophan levels and dietary nutrition may become a potential adjunctive treatment strategy for depression.

**Figure 3:**
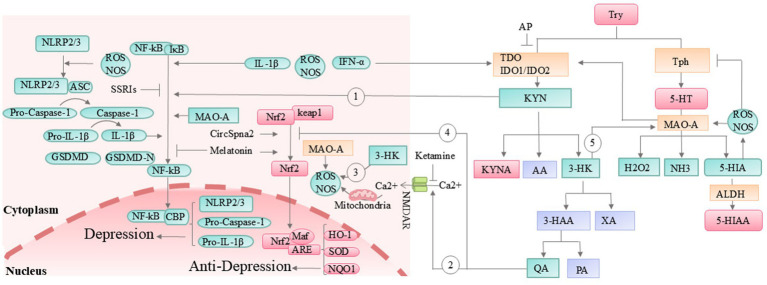
The kynurenine pathway and its association with depression. 3-HAA, 3-hydroxyanthranilic acid; 5-HIA, 5-hydroxyindoleacetaldehyde; AA, anthranilic acid; ALDH, aldehyde dehydrogenase; AP, allopurinol; ARE, antioxidant response element; ASC, apoptosis-associated speck-like protein containing a CARD; CBP, CREB-binding protein; GSDMD, gasdermin D; HK, 3-hydroxy-kynurenine; HO-1, heme oxygenase-1; IDO, indoleamine 2,3-dioxygenase; IFN-α, interferon alpha; IL-1β, interleukin-1 beta; Keap1, Kelch-like E-CH-associated protein 1; KYN, kynurenine; KYNA, kynurenic acid; MAO, monoamine oxidase; NF-κB, nuclear factor kappa-light-chain-enhancer of activated B cells; NLRP3, nuclear leucine-rich repeat protein 2/3; NMDAR, N-methyl-D-aspartate receptor; NOS, nitric oxide synthase; NQO1, NAD(P)H:quinone oxidoreductase 1; PA, picolinic acid; QA, quinolinic acid; SOD, superoxide dismutase; SSRIs, selective serotonin reuptake inhibitors; TD, tryptophan 2,3-dioxygenase; Tph, tryptophan hydroxylase; Try, tryptophan; XA, xanthurenic acid. Orange box: Key enzymes in tryptophan metabolic pathways. Green box: Factors that promote the onset and progression of depression. Red box: Factors that inhibit the onset and progression of depression. Purple box: Factors with minimal or unclear relevance to depression pathogenesis.

In addition, the metabolic imbalance of tryptophan is closely associated with depression. The most recognized metabolic pathways of tryptophan in the body include the kynurenine (95%) pathway, the 5-HT pathway, and the indole pathway (Li F. et al., 2024). The synthesis of kynurenine requires the catalytic action of key enzymes such as tryptophan 2,3-dioxygenase (TDO) and indoleamine 2,3-dioxygenase 1/2 (IDO1/IDO2) (Correia and Vale, 2022). Indole synthesis, on the other hand, is facilitated by the breakdown of tryptophan by colonic microbiota (Shaw et al., 2023). The dynamic balance between these pathways is essential for maintaining normal emotional functioning. Adding tryptophan to the diet reduces the levels of TDO, IDO, kynurenine, and IL-1 in the serum, while increasing the levels of tryptophan, 5-HT, and IL-22 (Li et al., 2022). However, when the body experiences chronic stress, gestational diabetes, lipopolysaccharide exposure, gut microbiota imbalance, interferon signaling activation, or increased pro-inflammatory factors, the activity of TDO or IDO1/IDO2 increases (Wichers et al., 2005; Correia and Vale, 2022; Shaw et al., 2023; Zhao et al., 2022), leading to the abnormal activation of the kynurenine pathway. The increased accumulation of kynurenine in the body activates the NF-kB-NLRP2-caspase1-IL-1β pathway (Figure 3, Gray loop 1), leading to an enhanced accumulation of inflammatory factors (Zhanga et al., 2020; Jiang et al., 2024). At the same time, as kynurenine metabolites such as quinolinic acid (QA) and 3-hydroxykynurenine (3-HK) continue to accumulate, the N-Methyl-D-Aspartate (NMDA) receptors are excessively activated (Figure 3, Gray loop 2), antioxidant factors like Nuclear Factor Erythroid 2-Related Factor 2 (Nrf2) are suppressed (Figure 3, Gray loop 3), and the generation of highly reactive free radicals occurs (Figure 3, Gray loop 4). Together, these processes contribute to excitotoxic damage to cells (Bansal et al., 2019; Tanaka et al., 2020). Since 3-HK can indirectly enhance MAO activity by generating highly reactive free radicals, under conditions where 5-HT and indole synthesis are limited, it further accelerates the degradation of 5-HT and potentially reduces the efficacy of SSRIs (Figure 3, Gray loop 5). These changes exacerbate neuroinflammation, the accumulation of neurotoxic metabolites, and imbalances in gut microbiota and BDNF, ultimately contributing to the development of depression (Correia and Vale, 2022; Katamanin et al., 2024; Myint and Halaris, 2022; Ryan et al., 2024) (Figure 3).

Based on these points, researchers have attempted to alleviate depressive symptoms by inhibiting the kynurenine pathway, achieving some positive results. The Jiang team confirmed that *Hypericum perforatum* L reduces kynurenine synthesis by increasing the abundance of the gut bacterium *Akkermansia muciniphila*, and successfully blocked the activation of the NF-kB-NLRP2-caspase1-IL-1β pathway, alleviating neuroinflammation and depressive-like symptoms in mice (Jiang et al., 2024). Compounds such as *berberine* (Wang Q. et al., 2022), isolated whey protein (Xia et al., 2023), and *crocetin* (Lin et al., 2022) have also been shown to improve depressive states in mice by downregulating IDO. The TDO inhibitor allopurinol also improved sepsis-induced depressive-like behavior in rats (Metzker et al., 2024). Ketamine, by antagonizing NMDA receptors (Liu R. et al., 2024) and reducing cellular oxidative stress damage, has gradually become one of the most effective new antidepressants. Meanwhile, *Clostridium butyricum* exerts its antidepressant effect by activating the 5-HT pathway (Zhang Z. W. et al., 2022). Additionally, circular RNA (CircSpna2), by preventing the ubiquitination of the antioxidant factor Nrf2 and increasing the expression of copper transport protein Atp7b, reduces oxidative stress and has shown a strong antidepressant effect in a traumatic brain injury mouse model (Du et al., 2024). This process may also be related to changes in IDO levels. It is worth mentioning that melatonin, the final product of the 5-HT pathway, not only activates the Nrf2 pathway but also inhibits NLRP3 activation, as well as the production of IL-1β and reactive oxygen species (ROS), ultimately alleviating lipopolysaccharide-induced depressive-like behaviors in mice (Arioz et al., 2019).

The relationship between tryptophan and depression discussed above is primarily related to the imbalance in tryptophan intake and metabolic pathways. Insufficient tryptophan intake or metabolic abnormalities can affect serotonin synthesis and increase the risk of depression. Supplementing tryptophan or its metabolites [such as 5-hydroxytryptophan (5-HTP)] can help improve depressive symptoms, but this involves various signaling pathways and effect targets, suggesting that depression is a complex disease influenced by multiple factors. Simply regulating tryptophan levels may not be sufficient as a primary treatment approach. Future research should further clarify the role of tryptophan metabolism in different types of depression and its underlying mechanisms, aiming to optimize nutritional intervention strategies and design related molecular-targeted drugs for adjunctive therapy.

### 4.3 Tph

Tph is the rate-limiting enzyme in serotonin synthesis, and due to its tissue specificity, it is often used as a marker for 5-HTergic neurons (Wang et al., 2024). In 1991, Craig et al. (1991) first identified the Tph1 gene through genetic mapping, locating it on chromosome 11 in the 11p15.3-p14 region, and confirmed its involvement in the synthesis of peripheral 5-HT. Subsequently, Walthe et al. (2003) discovered a new isoenzyme of tryptophan hydroxylase, Tph2, whose gene is located on human chromosome 12 at position 12q21, and it primarily participates in central 5-HT synthesis. As research into the gene structure and function progressed, certain single nucleotide polymorphism (SNP) mutations in these genes were found to be associated with psychiatric disorders. Carriers of these SNP alleles are often replaced, leading to heightened sensitivity to depression (Table 1). In adolescents with depression in northern China, the Tph2 gene locus rs11178997 AT genotype is more common (Liu W. et al., 2024). The AA genotype of TPH2 rs7305115 was significantly associated with suicidal behavior in major MDD patients from Shandong Province, China (Zhang et al., 2010). In patients from Minas Gerais, Brazil, the heterozygous C/T genotype of TPH2 SNP rs4565946 was negatively correlated with the risk of late-onset depression, while the homozygous A/A genotype of rs11179000 was positively correlated with this risk (Pereira et al., 2011). Zhang et al. (2005) discovered that when the allele G at position 1,463 of the Tph2 gene sequence is replaced by A, it leads to the substitution of arginine with histidine at position 441 of the Tph2 peptide chain, which reduces serotonin synthesis by 80%. Furthermore, SNP analysis of 87 patients with unipolar major depression from the Depression Research Center at Duke University revealed an increased frequency of the 1463A mutation. In patients with bipolar disorder (BD) from Germany and Russia, missense mutations at the TPH2 rs17110563 locus were also observed. These mutations were associated with reduced thermal stability and solubility of the enzyme, ultimately leading to decreased 5-HT synthesis and the development of bipolar disorder (Cichon et al., 2008). Additionally, allelic mutations may inhibit promoter function or induce selective exon splicing, altering Tph protein expression and activity, thereby further impeding 5-HT synthesis. For example, rs4570625 and rs11178997, located in the promoter region, can cause changes in the binding sites for transcription factors OCT-6 and POU3F2, leading to an increased susceptibility to depression (Walthe et al., 2003; Ma et al., 2015). However, no significant mutations in TPH2 rs4570625 were observed in Iranian patients with bipolar disorder. This discrepancy may be related to differences in genetic backgrounds and environmental factors across different populations (Hormozi et al., 2018). In addition, a repressive transcription factor related to the circadian rhythm, REV-ERBα, inhibits Tph2 expression by competitively binding to the Tph2 promoter region, which ultimately suppresses Tph2 transcription and induces depressive-like behaviors in mice (Park et al., 2024). The transcription factor REST, on the other hand, inhibits Tph expression by binding to the 5′ regulatory region RE-1, thereby suppressing Tph transcription (Arioz et al., 2019; Paresh et al., 2007). Increased methylation in the Tph promoter region leads to reduced Tph1/2 mRNA and protein expression (Chen et al., 2017), and has been confirmed to be associated with depression.

**Table 1:** The association between serotonin system gene mutations and mental disorders.

Gene	SNP	Mutation A1/A2	*p*-value	Disease	References
TPH1	rs1800532	A/C	0.04, 0.036, 0.022	BPD, SZ depression	Hormozi et al. (2018), Galaktionova et al. (2014), and Wigner et al. (2018)
	rs10488682	A/T	0.009	Depression	Wigner et al. (2018)
	rs623580	A/T	0.012	Depression	Wigner et al. (2018)
	rs1799913	A/C	0.042	Depression	Wigner et al. (2018)
TPH2	rs4570625	G/T	0.003, <0.001	Depression	Mandelli et al. (2012) and Ma et al. (2015)
	rs1023990	T/C	0.03	Depression	Mandelli et al. (2012)
	rs11178997	T/A	<0.05, 0.001	Depression	Liu W. et al. (2024) and Ma et al. (2015)
	rs11178998	A/G	0.0051	BPD	Cichon et al. (2008)
	rs7954758	A/G	0.0006	BPD	Cichon et al. (2008)
	rs7305115	A/G	<0.01	Depression	Zhang et al. (2010)
	rs120074175	A/G	0.008	Depression	Ma et al. (2015)
	rs11179000	T/A	0.025	Depression	Pereira et al. (2011)
	rs4565946	C/T	0.034	Depression	Pereira et al. (2011)
	rs17110563	C/T	0.0024	BPD	Cichon et al. (2008)
	rs4290270	T/A	0.002	Depression	Mei et al. (2018)
VMAT1 (SLC18A1)	rs988713	A/G	0.005	BPD1	Lohoff et al. (2006)
	rs952859	A/C	0.0007	AW	Dutta et al. (2016)
	rs2279709	G/C	0.038	BPD1	Lohoff et al. (2006)
	rs1390938	Thr136lle	0.003, 0.0006	BPD1, AW	Talkowski et al. (2008), Lohoff et al. (2013), Won et al. (2017), and Dutta et al. (2016)
	rs17215801	Phe84Ser	0.009	BPD	Lohoff et al. (2013)
	rs2270637	Thr98Ser	0.01	SZ	Lohoff et al. (2013)
	rs2270641	Thr4Pro	0.006	SZ	Lohoff et al. (2013)
VMAT2 (SLC18A2)	rs363338	C/T	0.031, 0.008, 0.043	OD, CI, SZ	Randesi et al. (2019), Zai et al. (2013), and Talkowski et al. (2008)
	rs363393	T/A	0.033, 0.011	SZ, CI	Zai et al. (2013) and Talkowski et al. (2008)
	rs363227	C/T	0.041, 0.027	SZ, CI	Zai et al. (2013) and Talkowski et al. (2008)
	rs393387	T/G	0.001	AD	Schwab et al. (2005)
	rs363226	C/G	0.043	CI	Myrga et al. (2016)
	rs363390	G/C	0.042	AD	Schwab et al. (2005)
AADC (DDC)	rs6592961	G/T	0.00047, 0.00067	Autism, ADHD	Toma et al. (2013) and Ribases et al. (2009)
	rs17733244	T/?	0.000097	Anxiety	Costas et al. (2010)
	rs921451	T/C	0.01, 0.02, 0.006	ND	Ma et al. (2005), Yu et al. (2006), and Loughlin et al. (2014)
	rs12718541	G/A	0.0002	ND	Yu et al. (2006)
	rs6592952	/	0.032	ADHD	Guan et al. (2009)
	rs11575542	G/A	0.0028	DD	Hack et al. (2011)
	rs11575461	C/T	0.00001	AD	Wei et al. (2012)
	rs2237457	C/T	0.00005	SZI	Li and Meltzer (2014)
	rs4947644	C/T	0.01	ND	Loughlin et al. (2014)
SERT (SLC6A4)	rs140700	G/?, ?/A	0.0000513, 0.037	Anxiety, depression	Costas et al. (2010) and Nyman et al. (2011)
	rs140701	C/T, G/A	0.0028, 0.048	PD, anxiety	Zou Z. et al. (2020) and Forstner et al. (2017)
	rs25528	A/C	0.003	Depression	Su et al. (2009)
	rs25531	A/G	<0.05	Anxiety, depression	Chang et al. (2017)
	rs3813034	T/G	0.0068	PD	Gyawali et al. (2010)
	rs2066713	T/C	<0.001, 0.044	SZ, depression	Vijayan et al. (2009) and Rucci et al. (2009)
	rs1042173	T/G	0.009	SZ	Schuch et al. (2016)
	rs2020934	C/T	0.013	Depression	Rucci et al. (2009)
	rs2020936	T/C	0.004	Depression	Su et al. (2009)
	rs6354	A/C	0.005	Depression	Su et al. (2009)
	rs6354, rs2020936	T/G, A/G	0.0062	Depression	Wray et al. (2009)

A1/A2, other allele/minor allele; AD, alcohol dependent; ADHD, attention-deficit/hyperactivity disorder; AW, alcohol withdrawal; BPD, bipolar affective disorder; Cl, cognitive impairment; DD, drug dependence; ND, nicotine dependence; OD, opioid dependence; PD, panic disorder; SZ, schizophrenia.

In addition to genetic regulation, Tph expression in the body is also influenced by various factors, such as substrate concentration (Koubi et al., 2001), hormones [cortisol (Betari et al., 2021), estrogen (Furukawa et al., 2024), prolactin (Goyvaerts et al., 2022), insulin (Zou X. H. et al., 2020), thyroid hormones (Melo et al., 2019)], catecholamines (Fitzpatrick, 2023), mitochondria (Park et al., 2021), inflammatory factors (Flamar et al., 2020), light cycles (Siemann et al., 2020), nutrition (Li K. et al., 2024), stress (Saroj et al., 2025), and genetics (Ping et al., 2019). Abnormal Tph expression in the brain has become an important topic in recent neuroscience research, particularly its association with depression. Researchers have conducted regional analysis of Tph expression in brain tissues from depression animal models, and found that Tph expression varies across different regions. Tph expression is often increased in the dorsal raphe nucleus (DRN) (Abumaria et al., 2006) and olfactory bulb (Chen et al., 2022), while decreased in regions such as the hippocampus (HP) (Lu et al., 2019), prefrontal cortex (PFC) (Lu et al., 2019), ventral tegmental area (VTA) (Galyamina et al., 2017), and hypothalamus (Shaif et al., 2018). Corresponding to these expression patterns, there are also differences in receptor activity across these regions. The DRN contains a large number of autoreceptors, forming a negative feedback loop for 5-HT signaling (Sharp and Barnes, 2020; Sun et al., 2022). Additionally, the 5-HTR2C are primarily located on Gamma-Aminobutyric Acid-ergic (GABAergic) interneurons in the DRN (Sharp and Barnes, 2020), which inhibit 5-HT signaling. In the PFC, the 5-HT2A receptors are mainly distributed and mediate excitatory effects. In the HP, 5-HTR1A, 5-HTR4, 5-HTR6, and 5-HTR7 are distributed, with most being postsynaptic receptors; except for 5-HTR1A, the rest exert excitatory effects (Sharp and Barnes, 2020). This signaling difference may explain the inconsistent changes in Tph expression across different brain regions in depression.

In terms of treatment, there appears to be a heightened interest in brain regions where Tph expression is decreased, with efforts focused on specifically activating these areas to alleviate depressive symptoms. The traditional Chinese medicine *Aurantii Fructus Immaturus-Carbonisata* has demonstrated significant antidepressant effects by upregulating Tph2 expression in the cortex of mice, thereby reducing immobility time in both the CUMS model and the reserpine-induced pain-depression dyad model (Li et al., 2023). Novel *isobenzofuran-1(3H)-one* derivatives have been shown to increase Tph2 expression in the HP, while *Dingzhi Xiaowan* enhances Tph expression in both the HP and PFC of rats, collectively contributing to its antidepressant effects (Tao et al., 2024; Dong et al., 2013). In addition, appropriate exercise, estrogen supplementation, gut microbiota improvement, and electroconvulsive therapy are also recognized as effective antidepressant strategies, all of which involve the regulation of Tph expression. Exercise not only increases HP Tph1 expression and the levels of neurotrophic factors such as nerve growth factor (NGF) and BDNF but also upregulates the expression of 5-HTR1A in the raphe nuclei, thereby alleviating depressive-like symptoms in rats (Hong et al., 2015; Shin et al., 2017). Additionally, animal models of depression induced by ovariectomy have successfully replicated depressive-like behaviors (Shaif et al., 2018), supporting the idea that estrogen deficiency is a potential mechanism underlying the pathogenesis of depression. Appropriate levels of estrogen activate protein kinase C (PKC) (Suganya et al., 2024), upregulating Tph2 while reducing MAO-A activity. This dual effect increases 5-HT synthesis and decreases its metabolism, thereby exerting significant resistance against depression (Farooqi et al., 2018). Regarding the gut microbiota, researchers have found that probiotics can enhance short-chain fatty acid synthesis, restore intestinal barrier function, reduce the absorption of bacteria and endotoxins, and alleviate inflammation-induced depressive symptoms (Aslam et al., 2020; Fang Y. et al., 2023). Moreover, gut-associated mucin-degrading *Akkermansia muciniphila* and *Clostridium butyricum* have been shown to upregulate intestinal Tph1 expression, leading to an increase in peripheral 5-HTP levels. These 5-HTP molecules cross the blood–brain barrier and serve as precursors for 5-HT synthesis in the central nervous system. By elevating central 5-HT levels, these mechanisms have successfully alleviated depressive-like behaviors in mice (Jiang et al., 2024; Zhang Z. W. et al., 2022). Meanwhile, electroconvulsive therapy (ECT) is also considered an effective treatment for depression (Yun and Kim, 2024). Koubi et al. (2001) found that acute ECT could increase Tph activity in rat brain tissue, which is thought to be a potential mechanism underlying its antidepressant effects. This may occur through the induction of heat shock protein (HSP) expression, such as HSP70 and HSP73 (Passarelli et al., 1994), which enhances Tph stability. However, further experimental studies are needed to validate this mechanism. Nevertheless, existing research is sufficient to establish Tph (particularly Tph2) as a critical molecule in the pathological mechanisms of depression. Its expression levels and activity, influenced by genetic polymorphisms, epigenetic modifications, intracellular signaling proteins, and the internal environment, mediate the regulation of 5-HT signaling and determine the susceptibility and severity of depression. Further exploration of the regulatory mechanisms of Tph and the development of personalized treatment approaches represent effective strategies for the prevention and management of depression.

### 4.4 AADC

AADC is another key enzyme required for the synthesis of monoamine neurotransmitters. It mediates the synthesis of serotonin, dopamine, norepinephrine, and epinephrine simultaneously (Roubertie et al., 2024). Moreover, due to its co-expression with angiotensin-converting enzyme 2, AADC is believed to be involved in the pathophysiological changes associated with COVID-19 (Attademo and Bernardini, 2020). The human gene encoding AADC is located on the short arm of chromosome 7, specifically at region 11. Clinically, a rare genetic disorder known as AADC deficiency is caused by pathogenic homozygous or compound heterozygous variants of the AADC gene (Roubertie et al., 2024; Rizzi et al., 2022). Patients with this condition suffer from severe deficiencies in monoamine neurotransmitters (Roubertie et al., 2024), resulting in a wide range of symptoms, including intellectual disability (Kanjia et al., 2024), motor control regulation disorders (Kanjia et al., 2024), mood disturbances (Baribeau et al., 2024), and autonomic nervous system dysfunction (Blau et al., 2023). In addition to AADC deficiency, the association between AADC gene SNPs and psychiatric disorders has received substantial research support, particularly in conditions such as schizophrenia, autism, and anxiety disorders. These genetic variations may influence the function of the enzyme and the synthesis of neurotransmitters, thereby affecting susceptibility to psychiatric diseases (Table 1). The T allele mutation at the AADC rs17733244 locus was found to be significantly associated with postpartum anxiety in Spanish women. Additionally, a combination of three SNPs (rs381901, rs2051684, and rs198183) in the protein kinase C, beta (PKCβ) gene sequence was closely linked to postpartum major depression (Costas et al., 2010). Ribases et al. (2009) conducted multiple gene SNP tests on 451 Spanish patients with Attention-Deficit/Hyperactivity Disorder (ADHD) and identified AADC rs6592961 as a continuous mutation affecting the entire lifespan, which is associated with susceptibility to ADHD in both children and adults. In a subsequent study, Toma et al. (2013) supplemented the mutation phenotype of rs6592961 and found that this mutation is also associated with susceptibility to autism in the Spanish population. It is worth mentioning that AADC appears to be an important candidate gene for nicotine dependence (ND), with several studies confirming this association. Among these, the AADC rs921451 and the T-G-T-G haplotype of rs921451-rs3735273-rs1451371-rs2060762 were initially found to be significantly associated with susceptibility to nicotine dependence (ND) in European-Americans and African-Americans (Ma et al., 2005). Subsequently, AADC rs12718541 (Yu et al., 2006) and rs4947644 (Loughlin et al., 2014) were also confirmed to be associated with ND. In addition to its role in susceptibility to mental disorders, AADC mutations may also be involved in the resistance mechanisms of psychiatric diseases. Li and Meltzer (2014) conducted a genome-wide association study on 174 Caucasian individuals with schizophrenia and found a significant association between the AADC SNP rs2237457 mutation and the patients’ resistance to olanzapine. Although the association between the AADC gene and psychiatric disorders has been extensively studied, direct research on its relationship with depression is still relatively limited. Future studies should further explore the role of AADC SNPs in depression, particularly regarding their interaction with neurotransmitter systems, influence on treatment response, and potential for personalized medicine.

In proteomics studies, high expression of AADC seems to be more closely associated with depression. Researchers have found a positive correlation between stress-induced depression and anxiety levels and the overexpression of AADC in populations susceptible to depression (Azadmarzabadi et al., 2018). Moreover, upregulation of AADC has also been observed in the HP region of rat models of depression (Jia et al., 2013). In VTA-clock knockout mice, AADC expression was increased, accompanied by depressive-like behavior (Mukherjee et al., 2010). In contrast, Kudryavtseva et al. (2017) found that while AADC expression increased in the VTA of depression and anxiety model mice, its expression decreased in the DRN (Galyamina et al., 2017). In addition, recent studies have shown conflicting results regarding the expression of AADC. Fang’s team suggested that during lipopolysaccharide (LPS)-induced depression, LPS inhibited the activity and expression of AADC by increasing plasma lipopolysaccharide-binding protein (LBP), ultimately leading to depressive-like phenotypes (Fang M. et al., 2023). Subsequently, Zhao et al. (2024) findings contradicted these results. They demonstrated that LPS exerts a pro-depressive effect by activating the RagA-mTOR-p70S6K pathway. However, during RagA activation, AADC expression in the prefrontal cortex was found to increase. From these studies, it is evident that, like Tph, AADC expression levels exhibit regional variability. However, the changes in their expression within the same brain region are exactly opposite. This contrast may reflect a compensatory mechanism by the body, attempting to salvage the abnormal 5-HT signaling pathway. Additionally, since AADC is involved in the synthesis of catecholamines, we must consider both types of neurons in the respective brain regions together. The VTA is rich in Dopamine (DA) neurons (Uliana et al., 2024), while the DRN is abundant in 5-HT neurons (Uliana et al., 2024; Feldman et al., 1997), and the HP and cortex can co-release both DA and 5-HT (Velazquez and Becerra, 2024; Wang M. et al., 2022). These regions, where AADC is involved in the regulation of different neurotransmitter systems, interact with one another and collectively contribute to the modulation of emotional function. In terms of treatment, Wang Q. et al. (2022) found that the traditional Chinese medicine *Berberine* increased AADC expression in the HP of depression model mice while downregulating the kynurenine metabolism pathway, exerting an antidepressant effect. Similarly, *Cordyceps sinensis*, another traditional Chinese medicine, also demonstrates antidepressant activity, and several active components extracted from it have been confirmed to act on AADC (Zhang X. et al., 2022). Additionally, insulin regulates the expression of AADC in the dorsolateral PFC and hippocampus (Zou X. H. et al., 2020), and activates the downstream target protein kinase B (AKT), which modulates dopamine transporter membrane expression, inhibits glycogen synthase kinase-3 (GSK3), and promotes the activation of the BDNF pathway, thereby reducing neuroinflammation and alleviating depressive symptoms (Jo et al., 2025). Significantly, BDNF can also enhance synaptic efficacy through dual modulation of presynaptic neurotransmitter release dynamics and postsynaptic receptor sensitivity via its high-affinity receptor tropomyosin receptor kinase B (TrkB), thereby inducing sustained potentiation of synaptic plasticity. This neurotrophic cascade ultimately mediates multiple neurobiological benefits, including robust antidepressant and anxiolytic effects, enhanced memory consolidation, promoted adult neurogenesis, and stimulated cerebral angiogenesis (Amato et al., 2020).

AADC simultaneously regulates serotonin and dopamine levels, and the deficiency of either neurotransmitter promotes the onset of depression. Therefore, AADC seems to play a crucial role in the pathophysiology and treatment of depression. Targeted treatment strategies for AADC, such as precursor supplementation, AADC inhibitors, and gene therapy, offer new approaches for antidepressant therapy. However, while AADC exerts powerful antidepressant effects by influencing multiple systems, it also presents new challenges for antidepressant therapies targeting AADC, including significant side effects, drug interactions, and individual differences. As a result, clinicians must consider a comprehensive and individualized approach when using such treatments. With further research into the specific mechanisms of AADC in different brain regions and pathological states, more precise and efficient treatments for depression are expected in the future.

### 4.5 Vmat

After tryptophan undergoes catalysis by two key enzymes, it is converted into 5-HT. The mature 5-HT is rapidly transported to the synaptic terminal for release or stored in vesicles through the action of Vmat. With the rapid development of contemporary neuroimaging techniques (Singh et al., 2024) and molecular biology techniques (Lohoff et al., 2013), the central role of Vmat in monoamine regulation has been gradually revealed. The currently identified Vmat include Vmat1 (SLC18A1) and Vmat2 (SLC18A2), which are located on chromosomes 8p21 and 10q25, respectively (Won et al., 2017). The gene sequences of the two are highly similar. Vmat1 is primarily found in peripheral tissues such as the adrenal medulla, intestines, and chromaffin cells (Erickson et al., 1996), whereas Vmat2 is predominantly concentrated in the monoaminergic neurons of the central nervous system (Lohoff et al., 2013; Peter et al., 1995) with some expression in the pancreas (Schäfer et al., 2014) and chromaffin cells (Erickson et al., 1996). They use the proton pump-driven electrochemical gradient across vesicular membranes to transport monoamine neurotransmitters into vesicles (Wandowski et al., 2016), preventing their degradation by intracellular metabolic systems. Genetic variations in the genes encoding Vmat may lead to abnormalities in vesicular transport and storage functions, disrupting the transmission of monoaminergic signals in mood regulation circuits, ultimately increasing the risk of emotional behaviors and psychiatric pathology (Won et al., 2017). This hypothesis has been confirmed in some case studies (Table 1). The rs1390938 variant is associated with bipolar disorder (characterized by both depressive and manic symptoms) and alcohol withdrawal reactions. This is caused by a mutation of the G allele to the A allele on the chromosome, resulting in the substitution of threonine with leucine at position 136 in the Vmat1 protein sequence (Won et al., 2017). The leucine-substituted Vmat1 exhibits higher transport activity (Lohoff et al., 2013), which leads to a weakened response to negative stimuli in brain regions such as the PFC and anterior cingulate cortex, which are involved in regulating emotional arousal, as well as enhanced responses in the amygdala (Lohoff et al., 2013) and decreased integrity of white matter (Won et al., 2017). These pathological changes may be the primary reasons why carriers of the A allele are more susceptible to emotional and anxiety disorders. Similarly, Lohoff et al. (2013) conducted DNA Sanger sequencing analysis on 4,023 European ancestry patients with BD and discovered a rare missense mutation at the rs17215801 site in the Vmat1 gene. This mutation results in the substitution of phenylalanine with serine at position 84 in the Vmat1 protein sequence, which increases the transport activity of the Vmat1 protein and is significantly associated with BP susceptibility. Another mutation, Arg138Leu (rs148468662), significantly reduces the transport activity of Vmat1 for 5-HT, but this mutation does not show a significant association with BP. Lohoff et al. (2006) also found that mutations at the rs988713 site in the promoter region and rs2279709 in intron 8 of the Vmat1 gene are associated with BP susceptibility in Europeans. Vmat2 SNPs, on the other hand, have been shown to be associated with psychiatric disorders other than depression. Specifically, the C-to-T substitution at the rs363338 site increases susceptibility to opioid dependence (OD) (Randesi et al., 2019), cognitive impairment (CI) (Zai et al., 2013), and schizophrenia (Talkowski et al., 2008). rs363393164 and rs363227155 are also linked to schizophrenia and CI, while rs393387 and rs363390 are associated with alcohol dependence (Schwab et al., 2005). Although these studies suggest an association between SNPs in Vmat1 and Vmat2 and psychiatric disorders, they may only represent a component of genetic susceptibility to mental illnesses. The exact mechanisms remain unclear, and further experimental evidence and clinical data are needed to validate their clinical application value.

Additionally, protein-level studies indicate that mood disorders, such as depression, may be closely related to dysfunction or abnormal expression levels of these transporters. Researchers have observed a reduction in Vmat2 levels in certain brain regions of depression animal models, such as the nucleus accumbens (NAc), VTA, and substantia nigra pars compacta (Schwartz et al., 2003). Furthermore, Vmat expression in the dorsolateral PFC shows sex differences; male depression patients exhibit lower expressions of both Vmat1 and Vmat2, whereas female depression patients show higher expression of Vmat2 and Tph2 (Bristow et al., 2021). These findings suggest that abnormalities in Vmat may be a potential pathophysiological mechanism of depression. Subsequently, researchers used gene editing technologies to directly manipulate Vmat, further confirming this hypothesis. Deficiency of Vmat significantly reduces 5-HT levels (Baronio et al., 2022). Vmat2 heterozygous mice exhibited reduced monoamine levels and displayed depressive-like behaviors (Fukui et al., 2007). Vmat2 knockout mice died shortly after birth (Wang et al., 1997), while Vmat2 knockdown zebrafish exhibited anxiety-like behaviors (Wang et al., 2016). Similarly, the use of selective Vmat2 inhibitors such as tetrabenazine (Connolly et al., 2024), fluoxetine (Correia et al., 2023), fenfluramine (Jay and Gingrich, 2001), and the non-selective Vmat inhibitor reserpine has induced depressive-like behaviors (Miguel Telega et al., 2024). Moreover, some of these adverse effects can be alleviated by antidepressant medications (Yohn et al., 2017; Yohn et al., 2015). However, the depressive effects of tetrabenazine remain controversial (Schultz et al., 2018). In contrast, carbamazepine metabolites (Rodrigues et al., 2023), lithium (Lohoff et al., 2006), valproates (Lohoff et al., 2006), melatonin (Stefanovic et al., 2016), and venlafaxine combined with melatonin (Tang et al., 2019) have been shown to increase Vmat levels, and most of these compounds exert antidepressant effects. The changes in Vmat expression or activity in depression models seem to be widely recognized. However, because Vmat exhibits high affinity for multiple monoamines, including 5-HT, norepinephrine, dopamine, and histamine, its alterations cannot be solely explained by 5-HT abnormalities. These changes may involve dysfunction across multiple neurotransmitter systems. Despite this, using imaging techniques and other auxiliary methods to detect changes in Vmat2 density and distribution in human brain regions (Singh et al., 2024) could provide new insights for the clinical diagnosis and treatment of depression.

### 4.6 SERT

#### 4.6.1 SERT and depression

SERT has a high affinity for 5-HT. It is primarily located at the presynaptic terminal, where it is responsible for reuptaking 5-HT from the synaptic cleft back into the cell, thereby terminating 5-HT signaling. SERT is functionally linked to Vmat (Sager and Torres, 2011), and together they regulate the homeostasis of the monoamine system in the body (Zheng et al., 2024). In the central nervous system, SERT is widely distributed across both cortical and subcortical regions (Zheng et al., 2024), with its density significantly decreasing with age (Yamamoto et al., 2002). As early as the 1980s, it was proposed that there was a certain association between SERT and depression. Researchers began attempting to treat depression by inhibiting SERT to increase extracellular 5-HT transmission, achieving satisfactory results (Wong et al., 1995). It wasn’t until the 1990s, with the successful cloning of genes encoding plasma membrane and Vmat, that the molecular structure and complete gene sequence of SERT were deciphered (Sager and Torres, 2011). SERT is composed of 630 amino acids and includes 12 transmembrane domains in its structure. The SERT gene, SLC6A4, is located on chromosome 17q11.2 and contains 14 exons and 13 introns. Malison et al. (1998) were the first to report a reduction in SERT density in the brainstem of patients with depression. Araki et al. (2024) also discovered that chronic stress leads to a decrease in SERT expression in the PFC, which in turn induces anxiety-like behavior. Additionally, Lesch et al. (1996) found that individuals susceptible to depression exhibited lower transcriptional activity of the SERT gene (SLC6A4), proposing that this was due to polymorphisms in the SLC6A4 promoter region. The short promoter (short allele, S) and long promoter (long allele, L) were identified, with these individuals often exhibiting the SS genotype. Subsequently, Caspi’s et al. (2003) study confirmed this finding.

This polymorphism in the SLC6A4 gene promoter region, particularly the 5-Hydroxytryptamine transporter linked polymorphic region (5-HTTLPR), along with related SNPs, has a significant impact on susceptibility to depression and other psychiatric disorders, as well as treatment response (Table 1). Allelic variations in rs25528 and rs2020936 were significantly associated with depression scores in Vietnamese males. Additionally, rs25528 was also linked to elevated levels of the inflammatory marker IL-6 (Su et al., 2009). The rs140700 mutation is not only associated with susceptibility to depression in the Finnish population (Nyman et al., 2011) but also positively correlated with the severity of postpartum anxiety in Spanish women (Costas et al., 2010). Additionally, the rs2066713 mutation is linked to the severity of schizophrenia in patients from southern India (Vijayan et al., 2009). Chang et al. (2017) found that Han Chinese men with the rs25531 mutation and the SS genotype in the promoter region were more likely to exhibit neuroticism and had higher anxiety/depression scores. Depressive phenotypes associated with SLC6A4 SNPs are also influenced by gender. Through 5-HTTLPR genotyping and questionnaire assessments in 222 Italian patients with unipolar major depression, he found that female patients with the “ll” and “ls” genotypes had significantly lower depression scores than males. Additionally, mutations rs2020942 and rs2066713 showed a depression association only in females (Rucci et al., 2009). Zou Z. et al. (2020) found a correlation between the rs140701 polymorphism and the risk of panic disorder in Han Chinese individuals, and he also observed that patients carrying the “S” genotype had a reduced sensitivity to sertraline treatment. Although these studies do not clarify the specific mechanisms between SNPs and the onset of depression, they highlight the possibility of different genetic variations among patients with depression. In clinical antidepressant treatment, doctors need to consider the genetic background of patients and choose appropriate antidepressant medications and treatment plans based on their genetic characteristics.

In addition to allele mutations, several transcription factors have been confirmed to participate in the transcriptional regulation of SLC6A4, such as Pet-1 (Goridis and Rohrer, 2002), Lmx1b (Song et al., 2014), ZFPM1 (Tikker et al., 2020), GATA2/3 (Haugas et al., 2016), cAMP (Baudry et al., 2019), and ATF4 (Baudry et al., 2019) (Table 2). Among them, Pet-1 has been shown to regulate serotonin across different stages of the life cycle. It recognizes specific sequences in the SLC6A4 promoter through its ETS domain, thereby modulating the transcription of the gene (Hendricks et al., 2003). Subsequent studies have also confirmed that Pet-1 participates in the transcriptional regulation of Tph2 and serotonin receptors, playing a multifaceted role in the development of depression. Current research indicates that deficiency of the PET-1 gene leads to multilevel dysfunction in the serotonin system, thereby inducing mood disorders and depressive-like behaviors. Hendricks, by knocking out Pet-1 in male mice, observed a significant loss of 5-HT neurons, accompanied by increased aggression (Hendricks et al., 2003). Later, Schaefer et al. (2009) found that Pet-1 knockout mice exhibited cognitive deficits and anxiety-like behaviors, along with increased activity disturbances and defensive behaviors. In recent research, Park et al. (2024) observed that knocking out Pet-1 in the DRN of mice led to a marked increase in depression-like behaviors at dawn. Additionally, the binding sites of PET-1 in the Tph2 promoter region are subject to competitive inhibition by the circadian nuclear receptor REV-ERBα. Targeted inhibition of REV-ERBα can rapidly and effectively alleviate depression-like phenotypes in mice. PET-1 is closely related to the development and function of serotonin neurons, making it a promising potential biomarker for the diagnosis of depression. The transcription factor Lmx1b plays a crucial role in the development and function of 5-HT neurons, particularly in regulating the formation and characteristics of these neurons. Song et al. (2014) specifically deleted Lmx1b in the DRN of mice, and found that although the 5-HT levels in the brain tissue of these mice were significantly reduced, with a downregulation of Tph2, Sert, and Vmat2 expression, the expression of Pet1, as well as the distribution and density of 5-HT neurons, did not show significant changes. This suggests that the influence of Lmx1b on 5-HT neurons is more prominent during development. On the other hand, the transcription factor ZFPM1 is more commonly associated with anxiety-like phenotypes (Tikker et al., 2020). Both GATA2 and GATA3 regulate the transcription of SERT and Tph2, playing a role in the development of 5-HT neurons (Haugas et al., 2016). Interestingly, phosphorylated STAT3 can reduce the expression of SERT in both colonic and brain tissues, inducing symptoms of irritable bowel syndrome (IBS) and depression-like behaviors (Shen et al., 2022; Kong et al., 2015). Shen et al. (2022) developed a new drug that blocks colonic STAT3 phosphorylation, ultimately alleviating IBS symptoms. It is likely that there are many unknown transcription factors associated with SERT in the body. Identifying and targeting these factors may become a new molecular strategy for the treatment of depression in the future.

**Table 2:** The association between SERT gene regulation, epigenetic modifications, and depression.

Related molecules	Expression of related molecules	SERT expression	Region	Related phenotypes
TF	Pet-1^−/−^	SERT^↓^TPH2^↓^	Systemic	Cognitive deficits (Schaefer et al., 2009)
		5-HTR1a^↓^		Anxiety (Hendricks et al., 2003; Schaefer et al., 2009; Liu et al., 2010)
		5-HTR1b^↓^	DRN	Aggression (Hendricks et al., 2003)
	Pet-1^−/−^	TPH2^↓^		Depression-like behaviors (Park et al., 2024)
	ZFPM1^cko^	SERT^↓^TPH2^↓^	DRVL	Anxiety-like behaviors (Tikker et al., 2020)
	GATA2^cko^	SERT^↓^TPH2^↓^	DRN	Defective serotonergic neuron development (Haugas et al., 2016)
	GATA3^cko^	SERT^↓^TPH2^↓^		
	Lmx1b^cko^	SERT^↓^TPH2^↓^	DRN	No obvious difference in density 5-HTergic neurons and distribution (Song et al., 2014)
	p-STAT3^Inhibitor^	SERT^↑^	Colon	Ameliorate IBS (Shen et al., 2022)
	p-STAT3	SERT^↓^	Brain	Depression (Kong et al., 2015)
DNA methylation	CpG 21^methylation↓^ CpG 25.26^methylation↓^		HTTLPR s/s	Depression (Lam et al., 2018)
	CpG 21^methylation↑^		5-HTTLPR l/l	Depression (Lam et al., 2018)
	CpG 1^methylation↑^ CpG 2^methylation↑^ CpG 3^methylation↑^		5-HTTLPR s/s	Poststroke depression (Kim et al., 2013)
	CpG 3^methylation↑^		Blood	Depression (Okada et al., 2014)
	CpG8^methylation↑^ CpG10^methylation↑^		Blood	Depression (Bakusic et al., 2020)
miRNA	miR-15↓ miR-16↓ miR-135a↓	SERT^↑^	PFC	Depression (Moya et al., 2013; Gheysarzadeh et al., 2018)
	miR-16^Inhibitor^	SERT^↑^	CSF	Depression (Song et al., 2015)
	miR-361↓	SERT^↑^	PASMCs	PASMCs proliferation (Zhang et al., 2020a)
	miR-18a-5p↓ miR-195-5p↓ miR-320-3p↓ miR-674-3p↓ miR-872-5p↓	SERT^↑^	PFC	Depression (Zurawek et al., 2017)
	miR-200a↑ miR-24↑	MicroRNA-200a^↑^ MiR-24^↑^	Intestinal mucosa	IBS (Hou et al., 2018; Liao et al., 2016)
lncRNA	lncRNA NONHSAG045500↑	SERT^↓^	FC	Ameliorate depression-like behaviors (Cui et al., 2023)
	LncRNA XIST^↑^	SERT^↓^	Rectal tissue	Ameliorate IBS (Zhang et al., 2020b)
	LncRNA H19^↑^	SERT^↑^	Ileum	Anbazhagan et al. (2023)
	LncRNA NEAT1^↑^	miR-320-3p^↓^	Hippocampal	Depression (Huang et al., 2024)
SIPs	nNOS-SERT↑	nNOS-SERT^↑^	DRN	Depression (Sun et al., 2022)
	FLOT1^KO^	SERT^↓^	Hippocampal	High sensitivity to the depressogenic effects (Reisinger et al., 2019; Zhan et al., 2023)
	syntaxin3	SERT^↓^	Caco-2 cells	(Motoike et al., 2021)

5-HTTLPR, serotonin-transporter-linked polymorphic region; DRN, dorsal raphe nucleus; DRVL, dorsal raphe ventrolateral; FLOT1, flotillin-1; GATA2, GATA binding protein 2; GATA3, GATA binding protein 3; Lmx1b, LIM homeobox transcription factor 1 beta; LncRNA, long non-coding RNA; miRNA, micro ribonucleic acid; nNOS, neuronal nitric oxide synthase; PFC, prefrontal cortex; IBS, irritable bowel syndrome; PASMCs, pulmonary artery smooth muscle cells; Pet-1, plasmacytoma-expressed transcript 1; p-STAT3, phosphorylated signal transducer and activator of transcription 3; SERT, serotonin transporter; SIPs, SERT interacting proteins; TF, transcription factor; ZFPM1, zinc finger protein multitype 1.

#### 4.6.2 The association between SLC6A4 methylation levels and depression

In the early 21st century, research into the epigenetic regulation of SERT became increasingly prominent. These studies revealed various transcriptional and epigenetic abnormalities, which may represent pathophysiological characteristics of MDD and potential pathogenic mechanisms. Among these studies, research on DNA methylation and gene expression regulation gradually uncovered how the epigenetic mechanisms of the SERT gene influence emotions, behaviors, and responses to environmental stressors. Multiple studies have shown that high methylation of the SLC6A4 promoter region is significantly associated with an increased risk of depression. High methylation may lead to dysfunction of the serotonin transporter, affecting neurotransmitter balance, and this methylation is often correlated with promoter polymorphisms. Okada et al. (2014) and Bakusic et al. (2020) separately extracted the SLC6A4 gene from the blood of depression patients and identified three cytosine-phosphate-guanine (CpG) sites with high methylation that were positively correlated with depression. Mohammadi’s et al. (2022) research also found elevated SLC6A4 methylation in MDD patients, and this high methylation was reduced after treatment with SERT inhibitors, along with improvements in depressive symptoms. Sanwald et al. (2021) found that there are gender differences in gene methylation levels, with female patients often exhibiting higher methylation levels, which are positively correlated with the severity of depression. However, recent studies have pointed out that abnormally low methylation in certain genetic regions is also involved in the development of depression. In his research, Lam et al. (2018) discovered that depression was significantly associated with decreased methylation levels of CpG sites 21 and 25–26 in the SLC6A4 promoter region, but this was only observed in individuals with the SS genotype. Conversely, high methylation of CpG 21 was more likely to occur in individuals with the LL genotype, leading to depressive symptoms. Another study also suggested that high methylation of CpG sites 1–3 in the SLC6A4 promoter region is associated with the SS genotype and depression (Kim et al., 2013). Overall, both high and low methylation levels can disrupt normal physiological and psychological processes, leading to the onset or progression of depression symptoms. Although these findings provide important insights into understanding the pathogenesis of depression and developing novel therapeutic strategies, the existing studies have relatively small sample sizes, and methylation patterns may vary due to factors such as ethnicity, geographic location, and sample sources (e.g., differences between brain tissue and peripheral blood samples). This could lead to some biases in the results. Furthermore, many studies are based on correlation analysis, making it difficult to establish clear causal relationships. It remains unclear whether high methylation precedes the onset of depression or if depression leads to changes in gene methylation status. Future research will require more in-depth longitudinal studies, animal model experiments, and further exploration of the exact molecular mechanisms, as well as the development of diagnostic and therapeutic approaches targeting the regulation of methylation status in depression.

#### 4.6.3 Non-coding RNA targeted regulation of SLC6A4

##### 4.6.3.1 MicroRNAs

In addition to the regulation of gene promoter regions, the transcription process of SLC6A4 also requires the participation of non-coding RNAs, which play an important role in the 5-HT signaling pathway. In the 1960s, non-coding RNAs first entered the scientific spotlight, and several microRNAs (miRNAs) that bind to the 3′ untranslated region (3′-UTR) of SLC6A4 were subsequently discovered (Table 2). These miRNAs regulate SERT expression by inhibiting or degrading the target mRNA. Through miRNA binding site prediction in the SLC6A4 3′-UTR region and immunohistochemical techniques, miR-15 and miR-16 were found to inhibit SERT expression in human placental choriocarcinoma JAR cells and rat raphe nucleus RN46A cells. Moreover, due to the close genomic proximity of miR-15 and miR-16, their regulation of SERT may also involve synergistic effects (Moya et al., 2013). MiR-16 expression was decreased in the cerebrospinal fluid of 36 Chinese depression patients compared to 30 healthy controls, as measured by ELISA in central and peripheral samples. Additionally, after injecting miR-16 inhibitors into the ventricles of rats, depressive-like behaviors were induced, while 5-HT levels in the CSF increased and SERT expression decreased (Song et al., 2015). Furthermore, serum analysis of 39 Iranian depression patients demonstrated significantly decreased levels of miR-16, miR-135a, and miR-1202 compared to healthy controls. Additionally, serum levels of miR-135a and miR-1202 demonstrated significant sensitivity and specificity in the diagnostic assessment of depression (Gheysarzadeh et al., 2018). Although this study did not investigate the effects of these non-coding RNAs on SERT, subsequent emerging research has addressed this gap and also revealed regional variations in miR-16 expression. Bright found that CUMS upregulated the expression of miR-16 and miR-135 in the rat ventromedial PFC (vmPFC) and NAc, but downregulated miR-16 in the raphe and SERT expression in the vmPFC (Bright and Akirav, 2023). Similarly, Zurawek’s et al. (2017) recent study discovered that CUMS not only induced depressive-like behaviors in rats but also downregulated the expression of miR-18a-5p, miR-195-5p, miR-320-3p, and miR-674-3p in the rat prefrontal cortex, while upregulating their expression in the VTA. This was accompanied by a decrease in SERT expression in the VTA, whereas no significant differences were observed in SERT expression in the PFC and DRN. Notably, since miR-195 can also target and regulate the expression of BDNF and glutamate receptors, it is believed to mediate the antidepressant effects of SSRIs (Guo et al., 2010). In addition, miR-26a-3p has been experimentally demonstrated to contribute to depression-like phenotypes in animal models through multiple pathways. Knockdown of miR-26a-3p in the hippocampal region of rats led to activation of the downstream PTEN/PI3K/Akt signaling pathway, inhibiting autophagy, promoting neuronal apoptosis, and neurodegeneration, ultimately inducing depressive-like behaviors in rats. On the other hand, overexpression of miR-26a-3p reversed the depressive-like phenotype in mice (Li et al., 2021). However, unfortunately, the study did not delve deeper into the relationship between miR-26a-3p and SERT signaling expression. Although miR-361-3p has been confirmed to inhibit SERT transcriptional regulation, research on it has primarily focused on pulmonary arterial hypertension (Zhang et al., 2020a). These findings are highly encouraging, although direct evidence establishing their causal role in depression remains limited. Nevertheless, the observed alterations in expression may be implicated in the pathological processes underlying depression.

These studies reveal the complex regulatory relationship between miRNAs and SERT, with specific pathological mechanisms varying across different brain regions. This variation may be related to the distinct molecular environments and functional requirements of neurons in different brain areas. The regional specificity of miRNA expression profiles opens up potential paths for personalized antidepressant therapies. Existing research has confirmed that some SSRIs exert their antidepressant effects by rescuing abnormal miRNA signaling. A classic antidepressant, fluoxetine, is one example, as it upregulates miR-16 to inhibit SERT expression (Baudry et al., 2010). Additionally, blood samples from patients with MDD treated with citalopram or sertraline show a significant increase in miR-16 expression and a corresponding decrease in SERT expression compared to untreated patients (Ahmadimanesh et al., 2023). These results suggest the essential role of miRNA regulation in antidepressant treatment. In addition to traditional medications, some potential antidepressants are also related to miRNAs. Portugalov et al. (2022) found that the fatty acid amide hydrolase (FAAH) inhibitor could reverse depression-like phenotypes induced by early life stress in rats and upregulate miR-135 in female rat medial PFC (mPFC) and miR-16 in male rat mPFC, without affecting SERT or 5-HTR1A expression. This phenomenon suggests that SERT may not be the only target of miR-135 and miR-16, and their targeting of other signaling pathways could also be an important part of the progression of depression. Cannabidiol has also been shown to improve depression-like behaviors in rats, but with an opposite regulatory mechanism. It downregulates miR-16 and miR-135 in the rat mPFC and upregulates miR-135 in the NAc, accompanied by an increase in 5-HTR1A expression in the vmPFC (Bright and Akirav, 2023). Furthermore, directly knocking out MicroRNA-32-5p can alleviate lipopolysaccharide-induced depression-like behaviors in mice, but through inhibiting astrocyte overactivation and upregulating glutamate receptor expression (Zhong et al., 2020). Although miRNA regulation of depression is not solely mediated by SERT, as SERT is a well-established target for antidepressants, gaining a deeper understanding of the interactions and mechanisms between miRNAs and SERT will still help improve the effectiveness of SSRIs and reduce side effects.

##### 4.6.3.2 Long non-coding RNAs

In addition, with the continuous development of the non-coding RNA field, lncRNAs have been shown to affect mRNA transcription in various ways. On one hand, long non-coding RNAs (lncRNAs) can directly act on DNA transcription initiation sites to either promote or inhibit mRNA production. LncRNAs can also alter mRNA splicing by binding to precursor mRNA, resulting in the generation of different splice variants. On the other hand, lncRNAs can function as competitive endogenous RNAs (ceRNAs), competing with mRNA for binding to miRNAs, thus relieving miRNA-mediated repression of mRNA and indirectly influencing mRNA translation. Some of these lncRNAs have been confirmed to be involved in the development of depression. Wang, by examining peripheral blood samples from 39 perinatal depression patients, found a reduction in the expression of six lncRNAs. After receiving mindfulness-integrated cognitive behavioral therapy, the expression of lncRNA NONHSAG004550 and NONHSAT125420 increased, accompanied by improvements in depressive symptoms. These lncRNAs have thus been proposed as potential new diagnostic biomarkers for perinatal depression (Wang et al., 2021). Cui replicated this result in subsequent experiments and further confirmed that the antidepressant effect of lncRNA NONHSAG045500 is achieved by activating the cAMP-PKA-CREB pathway to reduce SERT expression and increase 5-HT levels in the prefrontal cortex (Cui et al., 2023). Upregulation of lncRNA XIST also leads to the downregulation of SERT, but it primarily shows improvement in irritable bowel syndrome (Zhang et al., 2020b). LncRNA NEAT1 was found to be upregulated in depressed rat models, and it induces depressive-like behaviors by competitively binding to miR-320-3p, targeting the corticotropin-releasing hormone receptor 1. Silencing lncRNA NEAT1 improved the depressive-like behaviors in rats (Huang et al., 2024) (Table 2). Although the direct relationship between lncRNAs and SERT was not measured in this study, based on previous findings, we hypothesize that the antidepressant effect of silencing lncRNA NEAT1 is likely due to its relief of competitive inhibition on miR-320-3p, thus enhancing miR-320-3p’s inhibitory effect on SERT. Levels of LncRNA VLDLR-AS1 and LncRNA MALAT1 are considered to be closely associated with depression (Patel et al., 2024). Bella conducted an observational study involving 16 patients with BD and found that the expression of LncRNA MALAT-1, GAS-5, and miR-221-5p in serum was altered following antidepressant treatment (Bella et al., 2023). Maloum also analyzed blood samples from 50 patients with BD and found that, compared to healthy individuals, the expression levels of lncRNA SCAL1, RMST, and MEG3 were significantly reduced (Maloum et al., 2022). These phenomena suggest the important role of lncRNAs in the regulation of mood disorders; however, their exact targets and mechanisms remain unclear. They may be involved in the regulation of SERT or exert effects through other unknown signaling pathways. Nevertheless, it is undeniable that epigenetic regulation of genes is significantly associated with the onset of depression. These findings may offer novel biological markers for the diagnosis of depression, improving early diagnostic rates and providing new perspectives and research directions for understanding the molecular mechanisms of depression. This could also make it possible to enhance the efficacy of traditional antidepressants through specific non-coding RNA inhibitors, antisense oligonucleotides, or mimetics.

#### 4.6.4 The post-translational modifications of SERT and their connection to depression

Both *in vitro* and *in vivo* model studies have shown that the dynamic balance between SERT activity and expression requires complex chemical modifications. Normal post-translational modifications are a key mechanism for the fine regulation of its function. Hypophosphorylation, hypo-ubiquitination, hyper-glycosylation, and hyper-palmitoylation of the SERT protein may promote the pathological progression of depression by enhancing neurotransmitter uptake efficiency and membrane surface stability. Meanwhile, histone hyperserotonylation has been confirmed to be associated with the epigenetic regulatory mechanisms of MDD. These abnormal post-translational modifications not only provide a molecular biological basis for the targets and clinical efficacy of SSRIs but also lay a solid theoretical foundation for comprehensively understanding the functional regulation of SERT and its pathophysiological roles and therapeutic strategies in psychiatric disorders such as obsessive-compulsive disorder, depression, autism, and schizophrenia.

##### 4.6.4.1 SERT phosphorylation

**Figure 4:**
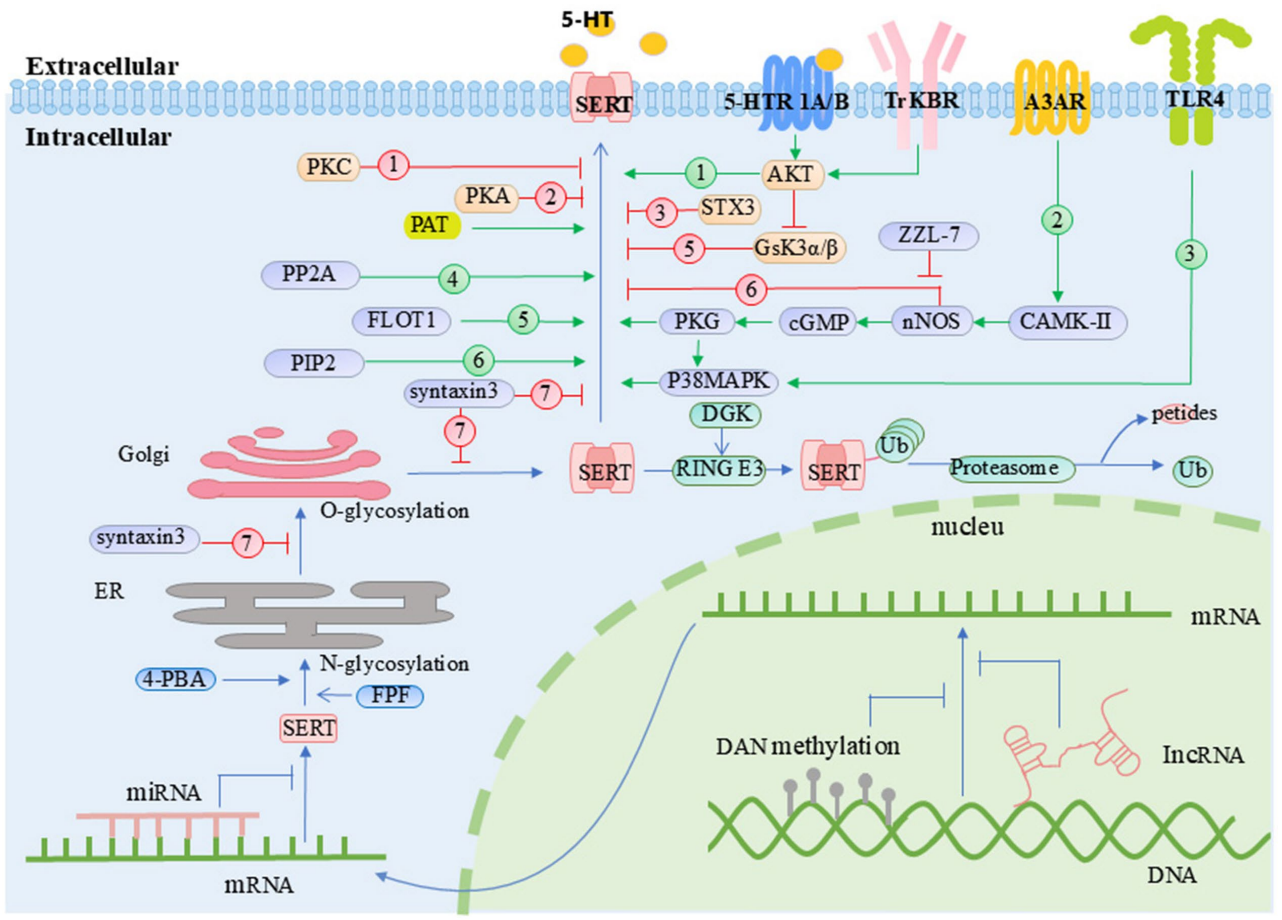
Regulatory mechanisms of SERT activity and membrane localization. 4-PBA, 4-phenylbutylate; A3AR, adenosine A3 receptor; AKT, protein kinase B; CAMK-II, calcium/calmodulin-dependent protein kinase II; cGMP, cyclic guanosine monophosphate; DGK, diacylglycerol kinase; ER, endoplasmic reticulum; FLOT1, flotillin-1; FPF, flubiroprofen; Gsk3α/β, glycogen synthase kinase 3 alpha/beta; Golgi, Golgi apparatus; MAGE-D1, melanoma antigen gene-D1; nNOS, neuronal nitric oxide synthase; PAT, palmitoyl acyltransferase; PKA, protein kinase A; PKC, protein kinase C; PKG, protein kinase G; PP2A, protein phosphatase 2A; RING E3, RING E3 ubiquitin ligase; SCAMP2, secretory carrier membrane protein 2; SERT, serotonin transporter; STX3, syntaxin 3; SSRIs, selective serotonin reuptake inhibitors; TLR4, Toll-like receptor 4; TrKBR, tropomyosin receptor kinase B receptor; Ub, ubiquitin. Orange box: Molecules that regulate SERT activity through post-translational modifications. Green box: Molecules involved in the ubiquitination pathway. Purple box: Molecules that modulate SERT activity via SIPs.

Phosphorylation is one of the most common post-translational modifications. By affecting protein stability and degradation rate, it indirectly influences its expression levels. Hypophosphorylation of SERT protein in the body is often accompanied by the emergence of depressive-like symptoms in experimental animals, and this process has been subsequently shown to require regulation by certain protein kinases. Akt induces a reduction in the basal phosphorylation level of SERT, accompanied by an increase in SERT’s 5-HT uptake capacity (Rajamanickam et al., 2015) (Figure 4, Green loop 1). In contrast, the downstream targets of Akt, GSK3α/*β*, attenuate SERT uptake capacity (Figure 4, Red loop 4) (Rajamanickam et al., 2015). Protein kinase C (PKC) is also recognized as a key factor regulating SERT function, and it may play an important role in depression by modulating the activity of SERT (serotonin transporter) and extracellular serotonin signaling levels. In his study, Takahiro found that stress-induced depression in mice led to a decrease in the levels of phosphorylated PKCβ1 and phosphorylated SERT in the PFC. However, activation of PKC increased SERT phosphorylation levels, reduced membrane surface SERT density, and enhanced extracellular serotonin signaling, ultimately alleviating the depressive-like symptoms in stressed mice (Ito et al., 2020) (Figure 4, Red loop 1). This effect of PKC closely resembles that of traditional SSRIs, both of which suppress SERT activity and expression, suggesting that PKC may be involved in the antidepressant mechanism of SSRIs. In addition, protein kinase A (PKA) also participates in regulating SERT activity. Activation of PKA promotes SERT phosphorylation and inhibits SERT’s membrane density and uptake function (Ramamoorthy et al., 2011) (Figure 4, Red loop 2). Besides catalyzing the phosphorylation of SERT itself to regulate serotonin signaling, the phosphorylation of transcription factors can also influence SERT activity. For example, when the transcription factor STAT3 is phosphorylated, it binds to the TT(N)AA site in the promoter region of SLC6A4, suppressing SERT transcription and increasing extracellular 5-HT levels. Inflammatory factor interleukin-6 activates STAT3 phosphorylation in the mouse brain, leading to a decrease in SERT expression and inducing depressive-like behaviors (Kong et al., 2015) (Figure 4, Red loop 3). These phenomena suggest that the regulation of SERT phosphorylation is inherently linked to depression and also reveal overlapping signaling pathways between the inflammatory hypothesis and the monoamine hypothesis of depression.

##### 4.6.4.2 SERT ubiquitination

In addition to phosphorylation modifications, the ubiquitin-proteasome system is also believed to regulate the stability of most cellular proteins. After a protein is tagged with ubiquitin, it is typically recognized by the proteasome, leading to its degradation. The ubiquitination status of SERT is a key regulatory factor in its proteasomal degradation, and the occurrence of depression is significantly associated with reduced levels of ubiquitinated SERT. The interaction between melanoma antigen gene-D1 (MAGE-D1) and RING E3 ubiquitin ligase enhances the ability of RING E3 to recognize and ubiquitinate target proteins. Mouri et al. (2012) found that knockdown of MAGE-D1 in the mouse mPFC resulted in reduced SERT ubiquitination levels, increased SERT expression, and depressive-like behaviors in the mice. This phenomenon could be reversed by the SSRI sertraline. Conversely, overexpression of MAGE-D1 in cells led to an increase in SERT ubiquitination levels, accompanied by a significant reduction in SERT activity and expression. Diacylglycerol kinase (DKG) can activate the PA/Praja-1/E3 ubiquitin-protein ligase pathway, increasing SERT ubiquitination and inducing its degradation. DGK knockout mice often exhibit compulsive behaviors (Lu et al., 2020). In clinical studies, Nakamura et al. (2024) found that patients with chronic orofacial pain comorbid with depression exhibited upregulation of total SERT and downregulation of ubiquitinated SERT in plasma. Duloxetine improved the depressive symptoms by increasing the ubiquitination of SERT, thereby reducing platelet SERT concentration. Additionally, Mouri et al. (2016) found that in patients with severe depression who were unresponsive to the antidepressant fluvoxamine, there was a significant reduction in the ubiquitination levels of peripheral blood lymphocyte SERT. This reduction led to insufficient degradation of SERT by proteasomes, resulting in elevated SERT expression and drug resistance in these patients. The natural degradation of SERT through ubiquitination provides more molecular targets for depression-targeted therapies. Combining ubiquitination regulators with traditional antidepressants could potentially generate a synergistic effect, enhancing the therapeutic efficacy of antidepressant treatments.

##### 4.6.4.3 SERT glycosylation

Glycosylation is another common post-translational modification, where carbohydrate groups are added to specific amino acid residues on proteins through enzymatic catalysis. The glycosylation process begins in the endoplasmic reticulum and continues in the Golgi apparatus. Hyper-glycosylation of SERT enhances its membrane expression and functional activity, which is crucial for maintaining normal serotonin reuptake. However, abnormal increases in this modification may regulate SERT transport efficiency and serve as one of the key molecular mechanisms underlying the development of depression. Both fluoxetine and 4-Phenylbutylate promote the correct glycosylation of SERT in the endoplasmic reticulum, which enhances its serotonin uptake activity and accelerates its transport to the plasma membrane (Hirakawa et al., 2022; Fujiwara et al., 2013). N-acetylglucosaminyltransferases (Mgat 1–5) are key enzymes in the Golgi apparatus responsible for the proper glycosylation of glycoproteins. Mgat1 is essential for normal growth and development, as Mgat1 knockout mice die by 8 weeks of age. Mgat2 knockout mice exhibit psychomotor disorders. In contrast, knockout of Mgat5 increases stress tolerance in mice and alleviates depressive-like behavior induced by chronic mild stress, but it also reduces sensitivity to SSRIs (Soleimani et al., 2008). Therefore, impaired glycosylation may serve as a predictive biomarker for antidepressant treatment response. Future efforts to repair or compensate for SERT glycosylation defects could potentially enhance patients’ responses to antidepressant medications.

##### 4.6.4.4 SERT palmitoylation

Palmitoylation is an increasingly recognized dynamic post-translational modification that, unlike ubiquitination, can enhance protein stability and prevent rapid degradation. Insufficient palmitoylation may lead to abnormal SERT localization, preventing it from effectively anchoring to the presynaptic membrane, thereby impairing 5-HT reuptake and disrupting neurotransmitter balance. Harada’s recent study found that the palmitoylation sites are located at SERT Cys-147 and Cys-155. Mutations at these sites result in reduced membrane expression of SERT and weakened 5-HT uptake ability. This suggests that palmitoylation plays a crucial role in regulating SERT function and stability, highlighting its potential importance in understanding neurobiological mechanisms related to mood disorders and antidepressant treatments (Harada et al., 2023). At the same time, Brown and Foster (2023) also observed the palmitoylation status of SERT and found that the irreversible palmitoyl acyltransferase inhibitor 2-bromopalmitate reduced SERT expression and transport activity. He noted that this phenomenon closely resembled the effects of the traditional antidepressant escitalopram, suggesting that palmitoylation may play a critical role in regulating SERT dynamics and transport capacity, potentially being the main mechanism by which escitalopram induces SERT internalization and exerts its antidepressant effects. Although SERT palmitoylation shows promise in depression research, it still faces many limitations. Current studies lack clinical validation, and the precise mechanisms by which palmitoylation regulates SERT function remain insufficiently understood. Future research will need to focus on further mechanistic studies, accumulating clinical data, and developing therapeutic strategies. These efforts will help elucidate the role of palmitoylation in depression and could drive its development as a novel therapeutic target.

##### 4.6.4.5 Serotonylation

It is worth noting that in addition to the various post-translational modifications of SERT itself, SERT can also influence the modification processes of other proteins. Serotonylation, which refers to the process by which serotonin, taken up by SERT into the cell, is bound to the glutamine residues of target proteins via transglutaminases, is one such modification (Pandya et al., 2017). This specific form of protein modification was not widely recognized and studied until the early 21st century. Among the various types of serotoninylation, histone serotoninylation is one of the most studied. It often occurs at specific sites of histone H3 in the cell nucleus, where it can influence gene expression and chromatin remodeling, thus playing a role in the regulation of various cellular processes, including those relevant to mood and depression. The level of serotoninylation on histone H3K4me3Q5ser in the DRN of the brain has been shown to positively correlate with aggressive behavior in rats (Meijer, 2023). In a recent study, Kachak et al. (2024) found that chronic stress leads to dysregulation of H3K4me3Q5ser serotoninylation in the DRN of mice, a change also observed in postmortem specimens from patients with MDD. Moreover, reducing H3K4me3Q5ser serotoninylation in stress model mice or treating them with fluoxetine improved both their depressive-like symptoms and the dysregulated H3K4me3Q5ser serotoninylation levels. This finding suggests that serotoninylation, through its regulation of the body’s gene expression program, plays a role in the onset of depression. Serotonylation of histone H3Q5 promotes the binding of the transcription factor TFIID initiation complex to promoters containing H3K4me3, playing a crucial role in regulating neuronal differentiation and maintaining neuronal plasticity (Jiang et al., 2021). In addition to histones, GTPases are another important target of serotoninylation. Pandya et al. (2017), in his research, found that overexpression of TGM2 led to a decrease in small GTPase Rac1 levels in the mouse prefrontal cortex, accompanied by depressive-like behaviors. This phenomenon suggests a potential link between Rac1 serotoninylation and depression. We hypothesize that the overexpression of TGM2 enhances Rac1 serotoninylation, leading to its decreased expression and ultimately inducing depressive-like behavior in mice. Serotonylation plays a significant role in the diagnosis and treatment of depression, serving as an important pathway for serotonin to regulate nervous system function.

#### 4.6.5 SERT interacting proteins

In addition to post-translational modifications, the uptake activity, membrane expression, and transport function of SERT are dynamically regulated by various SERT interacting proteins (SIPs). Understanding the regulatory patterns between SERT and SIPs can help us better comprehend the relationship between SERT expression and depression. Over 400 SIPs have been identified to date (Motoike et al., 2021). Recent studies suggest that some SIPs may play a role in the pathogenesis of depression. For example, neuronal nitric oxide synthase (nNOS) has been shown to physically interact with SERT, leading to a reduction in SERT’s uptake capacity and membrane expression (Sager and Torres, 2011) (Figure 4, Red loop 5). When SERT is inhibited by nNOS, extracellular 5-HT levels significantly increase. The excess 5-HT then enters the cell and activates nNOS, which produces more nitric oxide (NO). NO, in turn, further activates the cGMP/PKG pathway, enhancing SERT’s transport capacity and membrane expression (Zhong et al., 2012; Zhong et al., 2012) (Figure 4, Green loop 2). Their reciprocal interaction maintains normal 5-HT neurotransmission. Sun’s research found that CUMS enhances the coupling between nNOS and SERT in the mouse DRN, indirectly activating the 5-HT1A autoreceptor in the DRN, which induces depressive-like behaviors in mice. *ZZL-7* exerts antidepressant effects by blocking this interaction (Sun et al., 2022). In addition, the FLOT1 protein has been confirmed to interact with SERT. Compared to normal mice, FLOT1 knockout mice are more likely to exhibit depressive-like behaviors under chronic corticosterone stimulation, along with increased SERT expression in the hippocampal tissue (Reisinger et al., 2019). This suggests that FLOT1 may inhibit SERT activity through its interaction with SERT (Figure 4, Green loop 5). Effectively harnessing this effect could contribute to the development of novel antidepressant drugs. The phospholipid molecule phosphatidylinositol-4,5-bisphosphate (PIP2) drives SERT to the plasma membrane, enhancing its activity and promoting the uptake of extracellular 5-HT (Baudry et al., 2019) (Figure 4, Green loop 6). On the other hand, syntaxin 3 locks SERT within the endoplasmic reticulum and Golgi apparatus, preventing it from translocating to the plasma membrane to perform its normal function (Motoike et al., 2021) (Figure 4, Red loop 6). The SERT Gly56Ala mutation is associated with an autism phenotype. Robson discovered that this mutation activates p38 MAPK, leading to increased membrane stability of SERT, enhanced 5-HT uptake, and hypersensitivity of 5-HTR1A (Robson et al., 2018). Moreover, specific knockout of p38 MAPK in central 5-HT neurons prevented depressive-like behaviors in mice triggered by lipopolysaccharide stimulation and reduced 5-HTR1A sensitivity (Robson et al., 2018) (Figure 4, Green Loop 3). This phenomenon suggests that p38αMAPK activity is a major determinant of receptor and behavioral changes in SERT Ala56 mice and also reveals that alterations in p38αMAPK could be a potential pathogenic mechanism for depression. Quinlan et al. (2020) continued to analyze the SIPs in Gly56Ala mutant mice and found that, compared to the control group, 69 SIPs, including protein phosphatase 2A (PP2A), showed enhanced interaction with SERT (Figure 4, Green loop 4), while the interaction with 308 SIPs, including FLOT1, was weakened. The direct connection between these proteins and depression still requires further experimental validation in future studies. Since traditional antidepressants often have a certain latency before reaching their optimal effects [due to reasons such as 5-HTR1A hypersensitivity or compensatory mechanisms of low-affinity transporters (Bacq et al., 2012)], understanding the dynamic interactions between SIPs and SERT, and strategically utilizing or blocking SIPs’ modulation of SERT activity, could help us design new targeted drugs. This approach could improve the efficacy of traditional antidepressants and enhance the quality of life for patients.

## 5 Conclusion and future perspectives

Although growing evidence highlights the involvement of multiple complex biological processes in the pathophysiology of depression, the 5-HT system remains an integral and actively participating component in its development and progression. With advances in scientific technology, more mechanisms related to 5-HT in depression are emerging. These mechanisms collectively affect mood regulation through multiple pathways, including neurotransmitter levels, transport, and genetic polymorphisms. The development of modern antidepressant medications has benefited from the in-depth study of the 5-HT system. Research on the 5-HT system has not only deepened our biological understanding of depression but also provided crucial insights for developing more effective treatments. Future directions for research include exploring the complex network interactions between 5-HT and other neurotransmitters such as dopamine and norepinephrine; examining the dynamic changes in the 5-HT system across different emotional states and life environments; advancing gene-drug interaction studies to optimize precision treatment for depression; and integrating perspectives from neurobiology, psychology, and sociology to explore the role of the 5-HT system in cross-cultural and social contexts. Investigating gene-level mechanisms, neural network regulation, and personalized treatment approaches may further enhance the clinical application of the 5-HT system in depression treatment strategies.

## Glossary

3-HK: 3-Hydroxykynurenine
5-HIAA: 5-hydroxyindoleacetic acid
5-HT: 5-Hydroxytryptamine, serotonin
5-HTP: 5-Hydroxytryptophan
5-HTR: 5-Hydroxytryptamine Receptor
5-HTTLPR: 5-Hydroxytryptamine transporter linked polymorphic region
AADC: Aromatic L-amino acid decarboxylase
ADHD: Attention-deficit/hyperactivity disorder
AKT: Protein kinase B
ALDH: Aldehyde dehydrogenase
BD: Bipolar disorder
BDNF: Brain-derived neurotrophic factor
cAMP: Cyclic adenosine monophosphate
ceRNAs: Competitive endogenous RNAs
CI: Cognitive impairment
CpG: Cytosine-phosphate-guanine
CSF: Cerebrospinal fluid
CUMS: Chronic unpredictable mild stress
DA: Dopamine
DGK: Diacylglycerol kinase
DRN: Dorsal raphe nucleus
ECT: Electroconvulsive therapy
FAAH: Fatty acid amide hydrolase
GABA: Gamma-aminobutyric acid
GPCRs: G protein-coupled receptors
GR: Glucocorticoid receptor
GSK3: Glycogen synthase kinase-3
HP: Hippocampus
HPA: Hypothalamic-pituitary-adrenal
HSP: Heat shock protein
IBS: Irritable bowel syndrome
IDO: Indoleamine 2,3-dioxygenase
lncRNAs: Long non-coding RNAs
LBP: Lipopolysaccharide-binding protein
LPS: Lipopolysaccharide
MAGE-D1: Melanoma antigen gene-D1
MAO: Monoamine oxidase
MDD: Major depressive disorder
miRNAs: MicroRNAs
mPFC: Medial PFC
NAc: Nucleus accumbens
ND: Nicotine dependence
NGF: Nerve growth factor
NMDA: N-Methyl-D-aspartate
nNOS: Neuronal nitric oxide synthase
NO: Nitric oxide
Nrf2: Nuclear factor erythroid 2-related factor 2
OD: Opioid dependence
PFC: Prefrontal cortex
PKA: Protein kinase A
PKC: Protein kinase C
PP2A: Potein phosphatase 2A
QA: Quinolinic acid
ROS: Reactive oxygen species
SERT: Serotonin transporter
SNP: Single nucleotide polymorphism
SSRIs: Selective serotonin reuptake inhibitors
TDO: Tryptophan 2,3-dioxygenase
Tph: Tryptophan hydroxylase
Vmat: Vesicular monoamine transporter
vmPFC: Ventromedial PFC
VTA: Ventral tegmental area

## References

[ref1] Abumaria N. Rygula R. Hiemke C. Fuchs E. Reinecke U. H. Rüther E.. (2006). Effect of chronic citalopram on serotonin-related and stress-regulated genes in the dorsal raphe nucleus of the rat. Eur. Neuropsychopharmacol. 17, 417–429. doi: 10.1016/j.euroneuro.2006.08.009, PMID: 17182223

[ref2] Ahmad A. Rasheed N. Banu N. Palit G. (2010). Alterations in monoamine levels and oxidative systems in frontal cortex, striatum, and hippocampus of the rat brain during chronic unpredictable stress. Stress 13, 355–364. doi: 10.3109/10253891003667862, PMID: 20536337

[ref3] Ahmadimanesh M. Etemad L. Rad D. M. Ghahremani M. H. Mohammadpour A. H. Esfehani R. J.. (2023). Effect of citalopram and sertraline on the expression of miRNA-124, 132, and 16 and their protein targets in patients with depression. Iran. J. Basic Med. Sci. 26, 820–829. doi: 10.22038/IJBMS.2023.66496.14595, PMID: 37396946 PMC10311976

[ref4] Ahmed H. S. (2024). The multifaceted role of L-type amino acid transporter 1 at the blood-brain barrier: structural implications and therapeutic potential. Mol. Neurobiol. 62, 3813–3832. doi: 10.1007/s12035-024-04506-9 39325101

[ref5] Amato L. C. D. Speranza L. Volpicelli F. (2020). Neurotrophic factor BDNF, physiological functions and therapeutic potential in depression, neurodegeneration and brain cancer. Int. J. Mol. Sci. 21:7777. doi: 10.3390/ijms21207777, PMID: 33096634 PMC7589016

[ref6] Anbazhagan A. N. Griggs T. F. Sharma D. Kaur P. Priyamavada S. Jayawardena D.. (2023). LncRNA H19 regulates intestinal serotonin transporter (SERT) expression. Physiology 38:5734662. doi: 10.1152/physiol.2023.38.S1.5734662

[ref7] Ansari F. V. Tancredi A. N. Alvarenga A. F. F. Daigle M. Albert P. R. (2024). Rapid reorganization of serotonin projections and antidepressant response to 5-HT1A-biased agonist NLX-101 in fluoxetine-resistant cF1ko mice. Neuropharmacology 261:110132. doi: 10.1016/j.neuropharm.2024.110132, PMID: 39208980

[ref8] Anton A. H. Gennaro J. F. (1965). Norepinephrine and serotonin in the tissues and venoms of 2 pit vipers. Nature 208, 1174–1175. doi: 10.1038/2081174a0, PMID: 5870315

[ref9] Araki R. Kita A. Ago Y. Yabe T. (2024). Chronic social defeat stress induces anxiety-like behaviors via downregulation of serotonin transporter in the prefrontal serotonergic system in mice. Neurochem. Int. 174:105682. doi: 10.1016/j.neuint.2024.105682 38301899

[ref10] Arioz B. I. Tastan B. Tarakcioglu E. Tufekci K. U. Olcum M. Ersoy N.. (2019). Melatonin attenuates LPS-induced acute depressive-like behaviors and microglial NLRP3 inflammasome activation through the SIRT1/Nrf2 pathway. Front. Immunol. 10:1511. doi: 10.3389/fimmu.2019.01511, PMID: 31327964 PMC6615259

[ref11] Asberg M. Thorén P. Träskman L. Bertilsson L. Ringberger V. (1976). “Serotonin depression”—a biochemical subgroup within the affective disorders? Science 191, 478–480. doi: 10.1126/science.1246632, PMID: 1246632

[ref12] Aslam H. Green J. Jacka F. N. Collier F. Berk M. Pasco J.. (2020). Fermented foods, the gut and mental health: a mechanistic overview with implications for depression and anxiety. Nutr. Neurosci. 23, 659–671. doi: 10.1080/1028415X.2018.1544332, PMID: 30415609

[ref13] Attademo L. Bernardini F. (2020). Are dopamine and serotonin involved in COVID-19 pathophysiology? Eur. J. Psychiatry 35, 62–63. doi: 10.1016/j.ejpsy.2020.10.004, PMID: 33162632 PMC7598536

[ref14] Azadmarzabadi E. Haghighatfard A. Mohammadi A. (2018). Low resilience to stress is associated with candidate gene expression alterations in the dopaminergic signalling pathway. Psychogeriatrics 18, 190–201. doi: 10.1111/psyg.12312, PMID: 29423959

[ref15] Bacq A. Balasse L. Biala G. Guiard B. Gardier A. M. Schinkel A.. (2012). Organic cation transporter 2 controls brainnorepinephrine and serotonin clearance andantidepressant response. Mol. Psychiatry 17, 926–939. doi: 10.1038/mp.2011.87, PMID: 21769100

[ref16] Bagdy G. Makara G. B. (1994). Hypothalamic paraventricular nucleus lesions differentially affect serotonin-1A (5-HT1A) and 5-HT2 receptor agonist-induced oxytocin, prolactin and corticosterone responses. Endocrinology 134, 1127–1131. doi: 10.1210/endo.134.3.8119151, PMID: 8119151

[ref17] Baker G. B. Matveychuk D. MacKenzie E. M. Dursun S. M. Mousseau D. D. (2012). Monoamine oxidase inhibitors and neuroprotective mechanisms. Bull. Clin. Psychopharmacol. 22, 293–296. doi: 10.5455/bcp.20121030014051

[ref18] Bakusic J. Vrieze E. Ghosh M. Bekaert B. Claes S. Godderis L. (2020). Increased methylation of NR3C1 and SLC6A4 is associated with blunted cortisol reactivity to stress in major depression. Neurobiol. Stress 12:100272. doi: 10.1016/j.ynstr.2020.100272 PMC7739183 33344725

[ref19] Bansal Y. Singh R. Parhar I. Kuhad A. Soga T. (2019). Quinolinic acid and nuclear factor erythroid 2-related factor 2 in depression: role in neuroprogression. Front. Pharmacol. 10:452. doi: 10.3389/fphar.2019.00452 31164818 PMC6536572

[ref20] Baribeau D. A. Vorstman J. A. S. Pearson T. S. (2024). Selective serotonin reuptake inhibitor treatment post gene therapy for an ultrarare neurometabolic disorder (AADC deficiency). J. Am. Acad. Child Adolesc. Psychiatry 63, 571–573. doi: 10.1016/j.jaac.2024.01.015, PMID: 38460745

[ref21] Barnes J. M. Barnes N. M. Costall B. Ironside J. W. Naylor R. J. (1989). Identification and characterisation of 5-hydroxytryptamine 3 recognition sites in human brain tissue. J. Neurochem. 53, 1787–1793. doi: 10.1111/j.1471-4159.1989.tb09244.x 2809591

[ref22] Baronio D. Chen Y. C. Decker A. R. Enckell L. Fernández-López B. Semenova S.. (2022). Vesicular monoamine transporter 2 (SLC18A2) regulates monoamine turnover and brain development in zebrafish. Acta Physiol. 234:e13725. doi: 10.1111/apha.13725, PMID: 34403568

[ref23] Baudry A. Mouillet-Richard S. Schneider B. Launay J.-M. Kellermann O. (2010). miR-16 targets the serotonin transporter: a newfacet for adaptive responses to antidepressants. Science 329, 1537–1541. doi: 10.1126/science.1193692, PMID: 20847275

[ref24] Baudry A. Pietri M. Launay J. M. Kellermann O. Schneider B. (2019). Multifaceted regulations of the serotonin transporter: impact on antidepressant response. Front. Neurosci. 13:91. doi: 10.3389/fnins.2019.00091 30809118 PMC6379337

[ref25] Bella F. Muscatello M. R. A. D'Ascola A. Campo S. (2023). Gene expression analysis of nc-RNAs in bipolar and panic disorders: a pilot study. Genes 14:1778. doi: 10.3390/genes14091778, PMID: 37761918 PMC10530917

[ref26] Berger M. Gray J. A. Roth B. L. (2009). The expanded biology of serotonin. Annu. Rev. Med. 60, 355–366. doi: 10.1146/annurev.med.60.042307.110802, PMID: 19630576 PMC5864293

[ref27] Best J. Nijhout H. F. Reed M. (2010). Serotonin synthesis, release and reuptake in terminals: a mathematical model. Theor. Biol. Med. Model. 7:34. doi: 10.1186/1742-4682-7-34, PMID: 20723248 PMC2942809

[ref28] Betari N. Teigen K. Sahlholm K. Haavik J. (2021). Synthetic corticosteroids as tryptophan hydroxylase stabilizers, future. Med. Chem. 13, 1465–1474. doi: 10.4155/fmc-2021-0068, PMID: 34251270

[ref29] Bhatia S. C. Varma V. K. Amma M. P. K. (1978). A study of the relationship between urinary 5-hydroxyindoles and depressive states. Indian J. Psychiatry 20, 6–14.

[ref30] Bi Y. Huang N. Xu D. Wu S. Meng Q. Chen H.. (2024). Manganese exposure leads to depressive-like behavior through disruption of the Gln-Glu-GABA metabolic cycle. J. Hazard. Mater. 480:135808. doi: 10.1016/j.jhazmat.2024.135808 39288524

[ref31] Blau N. Pearson T. S. Kurian M. A. Elsea S. H. (2023). “Aromatic L-amino acid decarboxylase deficiency” in GeneReviews. eds. Adam M. P. Feldman J. Mirzaa G. M. (Seattle: University of Washington). 37824694

[ref32] Bright U. Akirav I. (2023). Cannabidiol modulates alterations in PFC microRNAs in a rat model of depression. Int. J. Mol. Sci. 24:2052. doi: 10.3390/ijms24032052, PMID: 36768376 PMC9953518

[ref33] Bristow G. C. Moul T. E. Lotesto K. Sodhi M. S. (2021). Sex differences in the transcription of monoamine transporters in major depression. J. Affect. Disord. 295, 1215–1219. doi: 10.1016/j.jad.2021.08.124, PMID: 34706435

[ref34] Brown C. R. Foster J. D. (2023). Palmitoylation regulates human serotonin transporter activity, trafficking, and expression and is modulated by escitalopram. ACS Chem. Neurosci. 14, 3431–3443. doi: 10.1021/acschemneuro.3c00319 37644775

[ref35] Brunetti L. Francavilla F. Leopoldo M. Lacivita E. (2024). Allosteric modulators of serotonin receptors: a medicinal chemistry survey. Pharmaceuticals 17. doi: 10.3390/ph17060695, PMID: 38931362 PMC11206742

[ref36] Bulut S. D. İspir G. Z. Bulut S. Aygün E. A. K. (2024). Comparison of the effect of selective serotonin and norepinephrine reuptake inhibitors on bone mineral density with selective serotonin reuptake inhibitors and healthy controls. J. Clin. Densitom. 28:101538. doi: 10.1016/j.jocd.2024.101538, PMID: 39536429

[ref37] Caspi A. Sugden K. Moffitt T. E. Taylor A. Craig I. W. Lee H.. (2003). Influence of life stress on depression: moderation by a polymorphism in the 5-HTT gene. Science 301, 386–389. doi: 10.1126/science.1083968, PMID: 12869766

[ref38] Castrén E. Rantamäki T. (2010). The role of BDNF and its receptors in depression and antidepressant drug action: reactivation of developmental plasticity. Dev. Neurobiol. 70, 289–297. doi: 10.1002/dneu.20758, PMID: 20186711

[ref39] Celada C. Pérez P. Álvarez J. Artigas F. (1992). Monoamine oxidase inhibitors phenelzine and brofaromine increase plasma serotonin and decrease 5-hydroxyindoleacetic acid in patients with major depression: relationship to clinical improvement. J. Clin. Psychopharmacol. 12, 309–315, PMID: 1282522

[ref40] Chang C. C. Chang H. A. Fang W. H. Chang T. C. Huang S. Y. (2017). Gender-specific association between serotonin transporter polymorphisms (5-HTTLPR and rs25531) and neuroticism, anxiety and depression in well-defined healthy Han Chinese. J. Affect. Disord. 207, 422–428. doi: 10.1016/j.jad.2016.08.055, PMID: 27788383

[ref41] Chen C. Y. Wang Y. F. Lei L. Zhang Y. (2024). MicroRNA-specific targets for neuronal plasticity, neurotransmitters, neurotrophic factors, and gut microbes in the pathogenesis and therapeutic. Prog. Neuro-Psychopharmacol. Biol. Psychiatry 136:111186. doi: 10.1016/j.pnpbp.2024.111186, PMID: 39521033

[ref42] Chen Y. Xu H. Zhu M. Liu K. Lin B. Luo R.. (2017). Stress inhibits tryptophan hydroxylase expression in a rat model of depression. Oncotarget 8, 63247–63257. doi: 10.18632/oncotarget.18780, PMID: 28968985 PMC5609917

[ref43] Chen G. Zhou S. Chen Q. Liu M. Dong M. Hou J.. (2022). Tryptophan-5-HT pathway disorder was uncovered in the olfactory bulb of a depression mice model by metabolomic analysis. Front. Mol. Neurosci. 15:965697. doi: 10.3389/fnmol.2022.965697, PMID: 36299862 PMC9589483

[ref44] Chojnacki C. Gąsiorowska A. Popławski T. Konrad P. Chojnacki M. Fila M.. (2023). Beneficial effect of increased tryptophan intake on its metabolism and mental state of the elderly. Nutrients 15:847. doi: 10.3390/nu15040847, PMID: 36839204 PMC9961537

[ref45] Chojnacki C. Popławski T. Chojnacki J. Fila M. Konrad P. Blasiak J. (2020). Tryptophan intake and metabolism in older adults with mood disorders. Nutrients 12:3183. doi: 10.3390/nu12103183, PMID: 33081001 PMC7603218

[ref46] Christenson J. G. Dairman W. Udenfriend S. (1970). Preparation and properties of a homogeneous aromatic-L-amino-acid decarboxylase from hog kidney. Arch. Biochem. Biophys. 141, 356–367. doi: 10.1016/0003-9861(70)90144-X, PMID: 4991409

[ref47] Cichon S. Winge I. Mattheisen M. Georgi A. Karpushova A. Freudenberg J.. (2008). Brain-specific tryptophan hydroxylase 2 (TPH2): a functional Pro206Ser substitution and variation in the 5′-region are associated with bipolar affective disorder. Hum. Mol. Genet. 17, 87–97. doi: 10.1093/hmg/ddm286, PMID: 17905754

[ref48] Connolly A. Wallman P. Dzahini O. Howes O. Taylor D. (2024). Meta-analysis and systematic review of vesicular monoamine transporter (VMAT-2) inhibitors in schizophrenia and psychosis. Psychopharmacology 241, 225–241. doi: 10.1007/s00213-023-06488-3, PMID: 38238580 PMC10805984

[ref49] Coppen A. (1967). The biochemistry of affective disorders. Br. J. Psychiatry 113, 1237–1264. doi: 10.1192/bjp.113.504.1237, PMID: 4169954

[ref50] Correia D. Bellot M. Prats E. Canela C. G. Moro H. Raldúa D.. (2023). Impact of environmentally relevant concentrations of fluoxetine on zebrafish larvae: from gene to behavior. Chemosphere 345:140468. doi: 10.1016/j.chemosphere.2023.140468, PMID: 37852383

[ref51] Correia A. S. Vale N. (2022). Tryptophan metabolism in depression: a narrative review with a focus on serotonin and kynurenine pathways. Int. J. Mol. Sci. 23, 8493–8510. doi: 10.3390/ijms23158493, PMID: 35955633 PMC9369076

[ref52] Costas J. Gratacòs M. Escaramís G. Santos R. M. Diego Y. D. García E. B.. (2010). Association study of 44 candidate genes with depressive and anxiety symptoms in post-partum women. J. Psychiatr. Res. 44, 717–724. doi: 10.1016/j.jpsychires.2009.12.012 20092830

[ref53] Craig S. P. Boularand S S. Darmon M. S. Mallet J. Craig I. W. (1991). Localization of human tryptophan hydroxylase (TPH) to chromosome 11p15.3—p14 by in situ hybridization. Cytogenet. Genome Res. 56, 157–159. doi: 10.1159/000133075, PMID: 2055111

[ref54] Cui X. Xu Y. Zhu H. Wang L. Zhou J. (2023). Long noncoding RNA NONHSAG045500 regulates serotonin transporter to ameliorate depressive-like behavior via the cAMP-PKA-CREB signaling pathway in a model of perinatal depression. J. Matern. Fetal Neonatal Med. 36:2183468. doi: 10.1080/14767058.2023.2183468, PMID: 36997170

[ref55] Dong X. Z. Li Z. L. Zheng X. L. Mu L. H. Zhang G. Liu P. (2013). A representative prescription for emotional disease, Ding-Zhi-Xiao-Wan restores 5-HT system deficit through interfering the synthesis and transshipment in chronic mild stress-induced depressive rats. J. Ethnopharmacol. 150, 1053–1061. doi: 10.1016/j.jep.2013.10.018, PMID: 24184266

[ref56] Du M. Fu J. Zhang J. Zhu Z. Huang X. Tan W.. (2024). CircSpna2 attenuates cuproptosis by mediating ubiquitin ligase Keap1 to regulate the Nrf2-Atp7b signalling axis in depression after traumatic brain injury in a mouse model. Clin. Transl. Med. 14:e70100. doi: 10.1002/ctm2.70100, PMID: 39581695 PMC11586089

[ref57] Duman R. S. Aghajanian G. K. (2012). Synaptic dysfunction in depression: potential therapeutic targets. Science 338, 68–72. doi: 10.1126/science.1222939, PMID: 23042884 PMC4424898

[ref58] Dutta N. Helton S. G. Schwandt M. Zhu X. Momenan R. Lohoff F. W. (2016). Genetic variation in the vesicular monoamine transporter 1 (VMAT1/SLC18A1) gene and alcohol withdrawal severity. Alcohol. Clin. Exp. Res. 40, 474–481. doi: 10.1111/acer.12991, PMID: 26876819

[ref59] Ejiohuo O. Bilska K. Narożna B. Skibinska M. Kapelski P. Węglarz M. D.. (2025). The implication of ADRA2A and AVPRIB gene variants in the aetiology of stress-related bipolar disorder. J. Affect. Disord. 368, 249–257. doi: 10.1016/j.jad.2024.09.072, PMID: 39278467

[ref60] Erickson J. D. Schafer M. K. Bonner T. I. Eiden L. E. Weihe E. (1996). Distinct pharmacological properties and distribution in neurons and endocrine cells of two isoforms of the human vesicular monoamine transporter. Proc. Natl. Acad. Sci. U.S.A. 93, 5166–5171. doi: 10.1073/pnas.93.10.5166, PMID: 8643547 PMC39426

[ref61] Erspamer V. Asero B. (1952). Identification of enteramine, the specific hormone of the enterochromaffin cell system, as 5-hydroxytryptamine. Nature 169, 800–801. doi: 10.1038/169800b0, PMID: 14941051

[ref62] Fähndrich E. Müller-Oerlinghausen B. Coper H. (1982). Longitudinal assessment of MAO-, COMT-, and DBH activity in patients with bipolar depression, international. Pharmacopsychiatry 17, 8–17. doi: 10.1159/000468552, PMID: 6979526

[ref63] Fang Y. Li Y. Liao X. Deng J. Wang Q. Liang J.. (2023). Corydalis yanhusuo polysaccharides ameliorate chronic stress-induced depression in mice through gut microbiota-derived short-chain fatty acid activation of 5-hydroxytryptamine signaling. J. Med. Food 26, 890–901. doi: 10.1089/jmf.2023.K.0050, PMID: 38010856

[ref64] Fang M. Li Y. Liao Z. Wang G. Cao Q. Li Y.. (2023). Lipopolysaccharide-binding protein expression is increased by stress and inhibits monoamine synthesis to promote depressive symptoms. Immunity 56, 620–634.e11. doi: 10.1016/j.immuni.2023.02.002, PMID: 36854305

[ref65] Farooqi N. A. I. Scotti M. Lew J. M. Botteron K. N. Karama S. McCracken J. T.. (2018). Role of DHEA and cortisol in prefrontal-amygdalar development and working memory. Psychoneuroendocrinology 98, 86–94. doi: 10.1016/j.psyneuen.2018.08.010 30121549 PMC6204313

[ref66] Fayyaz S. Wahab A. T. Siddiqui R. A. Choudhary M. I. (2024). Antidepressant sertraline hydrochloride inhibits the growth of HER2^+^ AU565 breast cancer cell line through induction of apoptosis and cell cycle arrest. Anti Cancer Agents Med. Chem. 24, 1038–1046. doi: 10.2174/0118715206304918240509111700, PMID: 38766835

[ref67] Feldman R. Meyer J. Quenzer L. (1997). Principles of neuropharmacology. Sunderland, MA: Sinauer Associates Inc.

[ref68] Fitzpatrick F. P. (2023). The aromatic amino acid hydroxylases: structures, catalysis, and regulation of phenylalanine hydroxylase, tyrosine hydroxylase, and tryptophan hydroxylase. Arch. Biochem. Biophys. 735:109518. doi: 10.1016/j.abb.2023.109518, PMID: 36639008

[ref69] Flamar A. L. Klose C. S. N. Moeller J. B. Mahlakõiv T. Bessman N. J. Zhang W.. (2020). DInter, leukin-33 induces the enzyme tryptophan hydroxylase 1 to promote inflammatory group 2 innate lymphoid cell-mediated immunity. Immunity 52, 606–619.e6. doi: 10.1016/j.immuni.2020.02.009, PMID: 32160524 PMC7218677

[ref70] Forstner A. J. Rambau S. Friedrich N. Kerstin L. Anne B. Elisabeth M.. (2017). Further evidence for genetic variation at the serotonin transporter gene SLC6A4 contributing toward anxiety. Psychiatr. Genet. 27, 96–102. doi: 10.1097/YPG.0000000000000171, PMID: 28272115

[ref71] Freis E. D. (1954). Mental depression in hypertensive patients treated for long periods with large doses of reserpine. New Engl. J. Med. 251, 1006–1008. doi: 10.1056/NEJM195412162512504, PMID: 13214379

[ref72] Fujiwara M. Yamamoto H. Miyagi T. Seki T. Tanaka S. Hide I.. (2013). Effects of the chemical chaperone 4-phenylbutylate on the function of the serotonin transporter (SERT) expressed in COS-7 cells. J. Pharmacol. Sci. 122, 71–83. doi: 10.1254/jphs.12194FP 23676312

[ref73] Fukui M. Rodriguiz R. M. Zhou J. Jiang S. X. Phillips L. E. Caron M. G.. (2007). Vmat2 heterozygous mutant mice display a depressive-like phenotype. J. Neurosci. 27, 10520–10529. doi: 10.1523/JNEUROSCI.4388-06.2007, PMID: 17898223 PMC2855647

[ref74] Furukawa M. Izumo N. Aoki R. Nagashima D. Ishibashi Y. Matsuzaki H. (2024). Behavioural changes in young ovariectomized mice via GPR30-dependent serotonergic nervous system. Eur. J. Neurosci. 60, 5658–5670. doi: 10.1111/ejn.16516, PMID: 39189108

[ref75] Gaddum J. H. Picarelli Z. P. (1957). Two kinds of tryptamine receptor. Br. J. Pharmacol. Chemother. 12, 323–328. doi: 10.1111/j.1476-5381.1957.tb00142.x, PMID: 13460238 PMC1509685

[ref76] Galaktionova D. I. Gareeva A. E. Khusnutdinova E. K. Nasedkina T. V. (2014). The association of polymorphisms in SLC18A1, TPH1 and RELN genes with risk of paranoid schizophrenia. Mol. Biol. 48, 546–555. doi: 10.1134/S0026893314030042, PMID: 25842846

[ref77] Galyamina A. G. Kovalenko I. L. Smagin D. A. Kudryavtseva N. N. (2017). Altered expression of neurotransmitters systems’ genes in the ventral tegmental area of depressive male mice: data of RNA-seq. Zh. Vyssh. Nerv. Deiat. Im. I P Pavlova 67, 113–128. doi: 10.7868/S0044467717010063 30695556

[ref78] Georgotas A. McCue R. E. Friedman E. Hapworth W. E. Kim O. M. Cooper T. B.. (1986). Relationship of platelet MAO activity to characteristics of major depressive illness. Psychiatry Res. 19, 247–256. doi: 10.1016/0165-1781(86)90118-6, PMID: 3809323

[ref79] Gheysarzadeh A. Sadeghifard N. Loghman A. Farahnaz P. Reza M. M. Hassan V.. (2018). Serum-based microRNA biomarkers for major depression miR-16, miR-135a, and miR-1202. J. Res. Med. Sci. 23:69. doi: 10.4103/jrms.JRMS_879_17, PMID: 30181751 PMC6116664

[ref80] Goridis C. Rohrer H. (2002). Specification of catecholaminergic and serotonergic neurons. Nat. Rev. Neurosci. 3, 531–541. doi: 10.1038/nrn871, PMID: 12094209

[ref81] Goyvaerts L. Iida A. S. Lemaire K. Veld P. Smolders I. Maroteaux L.. (2022). Normal pregnancy-induced islet beta cell proliferation in mouse models that are deficient in serotonin-signaling. Int. J. Mol. Sci. 23:15816. doi: 10.3390/ijms232415816, PMID: 36555462 PMC9779327

[ref82] Guan L. Wang B. Chen Y. Yang L. Li J. Qian Q.. (2009). A high-density single-nucleotide polymorphism screenof 23 candidate genes in attention deficit hyperactivitydisorder: suggesting multiple susceptibility genes among Chinese Han population. Mol. Psychiatry 14, 546–554. doi: 10.1038/sj.mp.4002139, PMID: 18180757

[ref83] Guo A. Y. Sun J. Jia P. Zhao Z. (2010). A novel microRNA and transcription factor mediated regulatory network in schizophrenia. BMC Syst. Biol. 4:10. doi: 10.1186/1752-0509-4-10 20156358 PMC2834616

[ref84] Gyawali S. Subaran R. Weissman M. M. Hershkowitz D. McKenna M. C. Talati A.. (2010). Association of a polyadenylation polymorphism in the serotonin transporter and panic disorder. Biol. Psychiatry 67, 331–338. doi: 10.1016/j.biopsych.2009.10.015, PMID: 19969287 PMC2980348

[ref85] Hack L. M. Kalsi G. Aliev F. Kuo P. H. Prescott C. A. Patterson D. G.. (2011). Limited associations of dopamine system genes with alcohol dependence and related traits in the Irish Affected Sib Pair Study of Alcohol Dependence (IASPSAD). Alcohol. Clin. Exp. Res. 35, 376–385. doi: 10.1111/j.1530-0277.2010.01353.x, PMID: 21083670 PMC3443636

[ref86] Hao S. Shi W. Liu W. Chen Q. Y. Zhuo M. (2023). Multiple modulatory roles of serotonin in chronic pain and injury-related anxiety. Front. Synaptic Neurosci. 15:1122381. doi: 10.3389/fnsyn.2023.1122381, PMID: 37143481 PMC10151796

[ref87] Harada K. Sho R. Takakura H. Yokoyama E. Koyama R. Adachi N.. (2023). S-Palmitoylation of the serotonin transporter promotes its cell surface expression and serotonin uptake. Biochem. Biophys. Res. Commun. 662, 58–65. doi: 10.1016/j.bbrc.2023.04.028, PMID: 37099811

[ref88] Harvey E. Silva D. Leatham N. Cameron M. Yeung A. (2017). Phenelzine protects brain mitochondrial function in vitro and in vivo following traumatic brain injury by scavenging the reactive carbonyls 4-hydroxynonenal and acrolein leading to cortical histological neuroprotection. J. Neurotrauma 34, 1302–1317. doi: 10.1089/neu.2016.4624, PMID: 27750484 PMC5385448

[ref89] Haugas M. Tikker L. Achim K. Salminen M. Partanen J. (2016). Gata2 and Gata3 regulate the differentiation of serotonergic and glutamatergic neuron subtypes of the dorsal raphe. Development 143, 4495–4508. doi: 10.1242/dev.136614 27789623

[ref90] He Y. Zhao B. Liu Z. Hu Y. Song J. Wu J. (2024). Individualized identification value of stress-related network structural-functional properties and HPA axis reactivity for subthreshold depression. Transl. Psychiatry 14:501. doi: 10.1038/s41398-024-03210-5, PMID: 39715743 PMC11666575

[ref91] Hendricks T. J. Fyodorov D. V. Wegman L. J. Lelutiu N. B. Pehek E. A. Yamamoto B.. (2003). Pet-1 ETS gene plays a critical role in 5-HT neuron development and is required for normal anxiety-like and aggressive behavior. Neuron 37, 233–247. doi: 10.1016/S0896-6273(02)01167-4, PMID: 12546819

[ref92] Hirakawa H. Taguchi K. Murakawa S. Asano M. Noguchi S. Kikkawa S.. (2022). Effects of flurbiprofen on the functional regulation of serotonin transporter and its misfolded mutant. J. Pharmacol. Sci. 148, 187–195. doi: 10.1016/j.jphs.2021.11.006, PMID: 34924125

[ref93] Hong Y. P. Lee H. C. Kim H. T. (2015). Treadmill exercise after social isolation increases the levels of NGF, BDNF, and synapsin I to induce survival of neurons in the hippocampus, and improves depression-like behavior. J. Exerc. Nutr. Biochem. 19, 11–18. doi: 10.5717/jenb.2015.19.1.11 PMC4424441 25960950

[ref94] Hormozi M. Zarei F. Rasouli A. Salimi S. Taji O. Nejad M. N. (2018). Association study of TPH1 (rs1800532) and TPH2 (rs4570625) polymorphisms in type 1 bipolar disorder in Iran. Gene Cell Tissue 5:e86109. doi: 10.5812/gct.86109

[ref95] Hou Q. Huang Y. Zhang C. Zhu S. Li P. Chen X.. (2018). MicroRNA-200a targets cannabinoid receptor 1 and serotonin transporter to increase visceral hyperalgesia in diarrhea-predominant irritable bowel syndrome rats. J. Neurogastroenterol. Motil. 24, 656–668. doi: 10.5056/jnm18037, PMID: 30347941 PMC6175558

[ref96] Hoyer D. Clarke D. E. Fozard J. R. Hartigal P. R. (1994). International Union of Pharmacology classification of receptors for 5-hydroxytryptamine (serotonin). Pharmacol. Rev. 46, 157–203. doi: 10.1016/S0031-6997(25)06783-3, PMID: 7938165

[ref97] Huang Y. Yao Y. S. Nan G. Mao Y. (2024). LncRNA NEAT1 inhibits neuronal apoptosis and induces neuronal viability of depressed rats via microRNA-320-3p/CRHR1 Axis. Neurochem. Res. 49, 2352–2363. doi: 10.1007/s11064-021-03508-6, PMID: 35075548

[ref98] Hung L. Y. Alves N. D. Colle A. D. Talati A. Najjar S. A. Bouchard V.. (2024). Intestinal epithelial serotonin as a novel target for treating disorders of gut-brain interaction and mood. Gastroenterology 168, 754–768. doi: 10.1053/j.gastro.2024.11.012 39672518 PMC12439035

[ref99] Hung N. L. Haia P. Y. T. Yuan J. M. Brand R. E. Pham T. V. Dao H. V.. (2024). Tryptophan intake and pancreatic cancer: findings from a case-control study. Eur. J. Cancer Prev. 33, 285–292. doi: 10.1097/CEJ.0000000000000864 38215023 PMC11156568

[ref100] Ito T. Hiramatsu Y. Uchida M. Yoshimi A. Mamiya T. Mouri A.. (2020). Involvement of protein kinase C beta1-serotonin transporter system dysfunction in emotional behaviors in stressed mice. Neurochem. Int. 140:104826. doi: 10.1016/j.neuint.2020.104826 32818536

[ref101] Jauhar J. S. Arnone D. Baldwin D. S. Bloomfield M. Browning M. Cleare A. J.. (2023). A leaky umbrella has little value: evidence clearly indicates the serotonin system is implicated in depression. Mol. Psychiatry 28, 3149–3152. doi: 10.1038/s41380-023-02095-y, PMID: 37322065 PMC10618084

[ref102] Jay A. Gingrich R. H. (2001). Dissecting the role of the serotonin system in neuropsychiatric disorders using knockout mice. Psychopharmacology 155, 1–10. doi: 10.1007/s002130000573 11374326

[ref103] Jia H. Feng Y. Liu Y. Chang X. Chen L. Zhang H.. (2013). Integration of ^1^H NMR and UPLC-Q-TOF/MS for a comprehensive urinary metabonomics study on a rat model of depression induced by chronic unpredictable mild stress. PLoS One 8:e63624. doi: 10.1371/journal.pone.0063624, PMID: 23696839 PMC3656962

[ref104] Jiang S. H. Wang Y. H. Hu L. P. Wang X. Li J. Zhang X. L.. (2021). The physiology, pathology and potential therapeutic application of serotonylation. J. Cell Sci. 134:jcs257337. doi: 10.1242/jcs.257337, PMID: 34085694

[ref105] Jiang Z. M. Wang F. F. Zhao Y. Y. Lu L. F. Jiang X. Y. Huang T. Q.. (2024). Hypericum perforatum L. attenuates depression by regulating *Akkermansia muciniphila*, tryptophan metabolism and NFκB-NLRP2-Caspase1-IL1β pathway. Phytomedicine 132:155847. doi: 10.1016/j.phymed.2024.155847 38996505

[ref106] Jo D. Choi S. Y. Ahn S. Y. Song J. (2025). IGF1 enhances memory function in obese mice and stabilizes the neural structure under insulin resistance via AKT-GSK3β-BDNF signaling. Biomed. Pharmacother. 183:117846. doi: 10.1016/j.biopha.2025.117846, PMID: 39805192

[ref107] Kachak A. A. Salvo G. D. Fulton S. L. Chan J. C. Farrelly L. A. Lepack A. E.. (2024). Histone serotonylation in dorsal raphe nucleus contributes to stress- and antidepressant-mediated gene expression and behavior. Nat. Commun. 15:5042. doi: 10.1038/s41467-024-49336-4, PMID: 38871707 PMC11176395

[ref108] Kanjia M. K. Jooste E. H. Illig M. Capps J. N. Eisner C. Fan S. Z.. (2024). Optimizing the anesthetic care of patients with aromatic L-amino acid decarboxylase deficiency. Paediatr. Anaesth. 35, 99–106. doi: 10.1111/pan.15025, PMID: 39435566 PMC11701947

[ref109] Kannen I. Bader M. Sakita J. Y. Uyemura S. A. Squire J. A. (2020). The dual role of serotonin in colorectal cancer. Trends Endocrinol. Metab. 31, 611–625. doi: 10.1016/j.tem.2020.04.008, PMID: 32439105

[ref110] Katamanin O. M. Tan I. J. Barry J. Jafferany M. (2024). Role of inflammation and cytokine dysregulation in depression in patients with inflammatory skin conditions. Am. J. Clin. Dermatol. 26, 35–43. doi: 10.1007/s40257-024-00905-9, PMID: 39623152

[ref111] Kim J. M. Stewart R. Kang H. J. Kim S. W. Shin I. S. Kim J.. (2013). A longitudinal study of SLC6A4 DNA promoter methylation and poststroke depression. J. Psychiatr. Res. 47, 1222–1227. doi: 10.1016/j.jpsychires.2013.04.010, PMID: 23702251

[ref112] Köhler C. A. Freitas T. H. Maes M. de Andrade N. Q. Liu C. S. Fernandes B. S.. (2017). Peripheral cytokine and chemokine alterations in depression: a meta-analysis of 82 studies. Acta Psychiatr. Scand. 135, 373–387. doi: 10.1111/acps.12698, PMID: 28122130

[ref113] Kong E. Sucic S. Monje F. J. Savalli G. Diao W. Khan D.. (2015). STAT3 controls IL6-dependent regulation of serotonin transporter function and depression-like behavior. Sci. Rep. 5:9009. doi: 10.1038/srep09009 25760924 PMC5390910

[ref114] Koubi D. Bezin L. Emard J. M. C. Gharib A. Bobillier P. Sarda N. (2001). Regulation of expression and enzymatic activities of tyrosine and tryptophan hydroxylases in rat brain after acute electroconvulsive shock. Brain Res. 905, 161–170. doi: 10.1016/S0006-8993(01)02524-0, PMID: 11423091

[ref115] Kudryavtseva N. N. Smagin D. A. Kovalenko I. L. Galyamina A. G. Vishnivetskaya G. B. Babenko V. N.. (2017). Serotonergic genes in the development of anxiety/depression-like state and pathology of aggressive behavior in male mice: RNA-seq data. Mol. Biol. 51, 251–262. doi: 10.1134/S0026893317020133 28537235

[ref116] Lam D. Ancelin M. L. Ritchie K. Poli R. F. Saffery R. Ryan J. (2018). Genotype-dependent associations between serotonin transporter gene (SLC6A4) DNA methylation and late-life depression. BMC Psychiatry 18:282. doi: 10.1186/s12888-018-1850-4, PMID: 30180828 PMC6122720

[ref117] Lemogne C. Vedrines C. O. Capuron L. Hoertel N. (2024). Inflammation and depressive mood. Joint Bone Spine 92:105832. doi: 10.1016/j.jbspin.2024.105832, PMID: 39719158

[ref118] Lesch K. P. Bengel D. Heils A. Sabol S. Z. Greenberg B. D. Petri S.. (1996). Association of anxiety-related traits with a polymorphism in the serotonin transporter gene regulatory region. Science 274, 1527–1531. doi: 10.1126/science.274.5292.1527, PMID: 8929413

[ref119] Li X. T. (2024). The involvement of K^+^ channels in depression and pharmacological effects of antidepressants on these channels. Transl. Psychiatry 14:411. doi: 10.1038/s41398-024-03069-6, PMID: 39358318 PMC11447029

[ref120] Li X. Dai E. Li M. Kong R. Yuan J. Li T.. (2023). *Aurantii fructus* immaturus carbonisata-derived carbon dots and their anti-depression effect. Front. Mol. Biosci. 10:1334083. doi: 10.3389/fmolb.2023.1334083 38259687 PMC10801177

[ref121] Li Y. Fan C. Wang L. Lan T. Gao R. Wang W.. (2021). MicroRNA-26a-3p rescues depression-like behaviors in male rats via preventing hippocampal neuronal anomalies. J. Clin. Invest. 131:e148853. doi: 10.1172/JCI148853, PMID: 34228643 PMC8363293

[ref122] Li F. Hu H. Ding L. Lu Z. Mao X. Wang R.. (2024). Integrated machine learning reveals the role of tryptophan metabolism in clear cell renal cell carcinoma and its association with patient prognosis. Biol. Direct 19:132. doi: 10.1186/s13062-024-00576-w, PMID: 39707545 PMC11662763

[ref123] Li J. Meltzer H. Y. (2014). A genetic locus in 7p12.2 associated with treatment resistant schizophrenia. Schizophr. Res. 159, 333–339. doi: 10.1016/j.schres.2014.08.018, PMID: 25223841

[ref124] Li K. Wei W. Xu C. Lian X. Bao J J. Yang S.. (2024). Prebiotic inulin alleviates anxiety and depression-like behavior in alcohol withdrawal mice by modulating the gut microbiota and 5-HT metabolism. Phytomedicine 135:156181. doi: 10.1016/j.phymed.2024.156181, PMID: 39488100

[ref125] Li Q. Zhou H. Ouyang J. Guo S. Zheng J. Li G. (2022). Effects of dietary tryptophan supplementation on body temperature, hormone, and cytokine levels in broilers exposed to acute heat stress. Trop. Anim. Health Prod. 54:164. doi: 10.1007/s11250-022-03161-3, PMID: 35435494

[ref126] Liao X. J. Mao W. M. Wang Q. Yang G. G. Wu W. J. Shao S. X. (2016). MicroRNA-24 inhibits serotonin reuptake transporter expression and aggravates irritable bowel syndrome. Biochem. Biophys. Res. Commun. 469, 288–293. doi: 10.1016/j.bbrc.2015.11.102, PMID: 26631964

[ref127] Lin S. Li Q. Xu Z. Chen Z. Tao Y. Tong Y.. (2022). Detection of the role of intestinal flora and tryptophan metabolism involved in antidepressant-like actions of crocetin based on a multi-omics approach. Psychopharmacology 239, 3657–3677. doi: 10.1007/s00213-022-06239-w, PMID: 36169685

[ref128] Lindseth G. Helland B. Caspers J. (2015). The effects of dietary tryptophan on affective disorders. Arch. Psychiatr. Nurs. 29, 102–107. doi: 10.1016/j.apnu.2014.11.008, PMID: 25858202 PMC4393508

[ref129] Liu W. Li G. Yang L. (2024). Association of non-suicidal self-injury with tryptophan hydroxylase 2 gene polymorphism and negative life events among adolescents with depression in northern China. Psychol. Res. Behav. Manag. 17, 2875–2883. doi: 10.2147/PRBM.S462835, PMID: 39104768 PMC11299724

[ref130] Liu R. Liu N. Ma L. Liu Y. Huang Z. Peng X.. (2024). Research progress on NMDA receptor enhancement drugs for the treatment of depressive disorder. CNS Drugs 38, 985–1002. doi: 10.1007/s40263-024-01123-x, PMID: 39379772

[ref131] Liu C. Maejima T. Wyler S. C. Casadesus G. Herlitze S. Deneris E. S. (2010). Pet-1 is required across different stages of life toregulate serotonergic function. Nat. Neurosci. 13, 1190–1198. doi: 10.1038/nn.2623, PMID: 20818386 PMC2947586

[ref132] Liu B. Zhang Y. Yang Z. Liu M. Zhang C. Zhao Y.. (2021). ω-3 DPA protected neurons from neuroinflammation by balancing microglia M1/M2 polarizations through inhibiting NF-κB/MAPK p38 signaling and activating neuron-BDNF-PI3K/AKT pathways. Mar. Drugs 19:587. doi: 10.3390/md19110587, PMID: 34822458 PMC8619469

[ref133] Lohoff F. W. Dahl J. P. Ferraro T. N. Arnold S. E. Gallinat J. Sander T.. (2006). Variations in the vesicular monoamine transporter 1 gene (VMAT1/SLC18A1) are associated with bipolar I disorder. Neuropsychopharmacology 31, 2739–2747. doi: 10.1038/sj.npp.1301196, PMID: 16936705 PMC2507868

[ref134] Lohoff F. W. Hodge R. Narasimhan S. Nall A. Ferraro T. N. Mickey B. J.. (2013). Functional genetic variants in the vesicular monoamine transporter 1 modulate emotion processing. Mol. Psychiatry 19, 129–139. doi: 10.1038/mp.2012.193, PMID: 23337945 PMC4311877

[ref135] Loomer H. P. Saunders J. C. Kline N. S. (1957). A clinical and pharmacodynamic evaluation of iproniazidas a psychic energizer. Psychiatr. Res. Rep. Am. Psychiatr. Assoc. 8, 129–141, PMID: 13542681

[ref136] Loughlin J. O. Sylvestre M. P. Labbe A. Low N. C. Roy-Gagnon M. H. Dugas E. N.. (2014). Genetic variants and early cigarette smoking and nicotine dependence phenotypes in adolescents. PLoS One 9:e115716. doi: 10.1371/journal.pone.0115716, PMID: 25545355 PMC4278712

[ref137] Lovenberg W. Jequier E. Sjoerdsma A. (1967). Tryptophan hydroxylation: measurement in pineal gland, brainstem, and cortex. Science 155, 217–219. doi: 10.1126/science.155.3759.217, PMID: 6015530

[ref138] Lu Q. Mouri A. Yang Y. Kunisawa K. Teshigawara T. Hirakawa M.. (2019). Chronic unpredictable mild stress-induced behavioral changes are coupled with dopaminergic hyperfunction and serotonergic hypofunction in mouse models of depression. Behav. Brain Res. 372:112053. doi: 10.1016/j.bbr.2019.112053, PMID: 31288060

[ref139] Lu Q. Murakami C. Murakami Y. Hoshino F. Asami M. Usuki T.. (2020). 1-Stearoyl-2-docosahexaenoyl-phosphatidic acid interact swith and activates Praja-1, the E3 ubiquitin ligase acting on the serotonin transporter in the brain. FEBS Lett. 594, 1787–1796. doi: 10.1002/1873-3468.13765, PMID: 32134507

[ref140] Ma J. Z. Beuten J. Payne T. J. Dupont R. T. Elston R. C. Li M. D. (2005). Haplotype analysis indicates an association between the DOPA decarboxylase (DDC) gene and nicotine dependence. Hum. Mol. Genet. 14, 1691–1698. doi: 10.1093/hmg/ddi177, PMID: 15879433

[ref141] Ma J. Xiao H. Yang Y. Cao D. Wang L. Yang X.. (2015). Interaction of tryptophan hydroxylase 2 gene and life events in susceptibility to major depression in a Chinese Han population. J. Affect. Disord. 188, 304–309. doi: 10.1016/j.jad.2015.07.041, PMID: 26386440

[ref142] Malison R. T. Price L. H. Berman R. Dyck C. H. V. Pelton G. H. Carpenter L.. (1998). Reduced brain serotonin transporter availability in major depression as measured by [^123^I]-2 beta-carbomethoxy-3 beta-(4-iodophenyl)tropane and single photon emission computed tomography. Biol. Psychiatry 44, 1090–1098. doi: 10.1016/S0006-3223(98)00272-8, PMID: 9836013

[ref143] Maloum Z. Taheri M. Ghafouri-Fard S. Farsani Z. S. (2022). Significant reduction of long non-coding RNAs expression in bipolar disorder. BMC Psychiatry 22:256. doi: 10.1186/s12888-022-03899-y, PMID: 35410190 PMC9004165

[ref144] Mandelli L. Antypa N. Nearchou F. A. Vaiopoulos C. Stefanis C. N. Serretti A.. (2012). The role of serotonergic genes and environmental stress on the development of depressive symptoms and neuroticism. J. Affect. Disord. 142, 82–89. doi: 10.1016/j.jad.2012.03.047, PMID: 22868061

[ref145] Mao B. Liu S. Zhu S. Wu F. Yuan W. Yan Y.. (2024). The janus face of serotonin: regenerative promoter and chronic liver disease aggravator. Heliyon 10:e30703. doi: 10.1016/j.heliyon.2024.e30703, PMID: 38756588 PMC11096747

[ref146] Mauler M. Bode C. Duerschmied D. (2016). Platelet serotonin modulates immune functions. Hamostaseologie 36, 11–16. doi: 10.5482/HAMO-14-11-0073, PMID: 25693763

[ref147] Mawe G. M. Kenton M. Camilleri M. (2023). Overview of the enteric nervous system. Semin. Neurol. 43, 495–505. doi: 10.1055/s-0043-1771466, PMID: 37562453

[ref148] Mei F. Wu Y. Wu J. (2018). The relationship between tryptophan hydroxylase-2 gene with primary insomnia and depressive symptoms in the Han Chinese population. Balkan Med. J. 35, 412–416. doi: 10.4274/balkanmedj.2017.1406, PMID: 29952309 PMC6251380

[ref149] Meijer M. (2023). The potential role of histone serotonylation in stress induced aggressive behaviour. Radboud Repository, 295–315.

[ref150] Melo R. L. Souza R J. S. Conceição R. Albuquerque J. M. L. Rodrigues N. C. Marinho B. G.. (2019). Prenatal thyroxine treatment promotes anxiolysis in male Swiss mice offspring, prenatal thyroxine treatment promotes anxiolysis in male Swiss mice offspring. Horm. Behav. 108, 10–19. doi: 10.1016/j.yhbeh.2018.12.008 30576638

[ref151] Metzker K. L. L. Mathias K. Machado R. S. Bonfante S. Joaquim L. Goulart M.. (2024). Amelioration of neurochemical alteration and memory and depressive behavior in sepsis by allopurinol, a tryptophan 2,3-dioxygenase inhibitor. CNS Neurol. Disord. Drug Targets 23, 1499–1515. doi: 10.2174/0118715273282363240415045927, PMID: 38712373

[ref152] Meyer H. Ginovart N. Boovariwala A. Sagrati S. Hussey D. Garcia A.. (2006). Elevated monoamine oxidase A levels in the brain an explanation for the monoamine imbalance of major depression. Arch. Gen. Psychiatry 3, 1209–1216. doi: 10.1001/archpsyc.63.11.1209 17088501

[ref153] Miguel Telega L. Berti R. Blazhenets G. Domogalla L. C. Steinacker N. Omrane M. A.. (2024). Reserpine-induced rat model for depression: Behavioral, physiological and PET-based dopamine receptor availability validation. Prog. Neuropsychopharmacol. Biol. 133:111013. doi: 10.1016/j.pnpbp.2024.111013 38636702

[ref154] Miller A. H. Raison C. L. (2016). The role of inflammation in depression: from evolutionary imperative to modern treatment target. Nat. Rev. Immunol. 16, 22–34. doi: 10.1038/nri.2015.5, PMID: 26711676 PMC5542678

[ref155] Mohammadi S. Pajooh A. B. Ahmadimanesh M. Ghazi-Khansari M. Moallem S. A. Hosseini R.. (2022). Evaluation of DNA methylation in BDNF, SLC6A4, NR3C1 and FKBP5 before and after treatment with selective serotonin-reuptake inhibitor in major depressive disorder. Epigenomics 14, 1269–1280. doi: 10.2217/epi-2022-0246 36377555

[ref156] Moncrieff J. Cooper R. E. Stockmann T. Amendola S. Hengartner M. P. Horowitz M. A. (2023). The serotonin theory of depression: a systematic umbrella review of the evidence. Mol. Psychiatry 28, 3243–3256. doi: 10.1038/s41380-022-01661-0, PMID: 35854107 PMC10618090

[ref157] Motoike S. Taguchi K. Harada K. Asano M. Hide I. Tanaka S.. (2021). Syntaxin 3 interacts with serotonin transporter and regulates its function. J. Pharmacol. Sci. 145, 297–307. doi: 10.1016/j.jphs.2021.01.007, PMID: 33712280

[ref158] Mouri A. Ikeda M. Koseki T. Iwata N. Nabeshima T. (2016). The ubiquitination of serotonin transporter in lymphoblasts derived from fluvoxamine-resistant depression patients. Neurosci. Lett. 617, 22–26. doi: 10.1016/j.neulet.2016.01.064, PMID: 26845564

[ref159] Mouri A. Sasaki A. Watanabe K. Sogawa C. Kitayama S. Mamiya T.. (2012). MAGE-D1 regulates expression of depression-like behavior through serotonin transporter ubiquitylation. J. Neurosci. 32, 4562–4580. doi: 10.1523/JNEUROSCI.6458-11.2012, PMID: 22457503 PMC6622051

[ref160] Moya P. R. Wendland J. R. Salemme J. Fried R. L. Murphy D. L. (2013). miR-15a and miR-16 regulate serotonin transporter expression in human placental and rat brain raphe cells. Int. J. Neuropsychopharmacol. 16, 621–629. doi: 10.1017/S1461145712000454, PMID: 22564678

[ref161] Mukherjee S. Coque L. Cao J. L. Kumar J. Chakravarty S. Asaithamby A.. (2010). Knockdown of clock in the ventral tegmental area through RNA interference results in a mixed state of Mania and depression-like behavior. Biol. Psychiatry 68, 503–511. doi: 10.1016/j.biopsych.2010.04.031, PMID: 20591414 PMC2929276

[ref162] Myint A. M. Halaris A. (2022). Imbalances in kynurenines as potential biomarkers in the diagnosis and treatment of psychiatric disorders. Front. Psychiatry 13:913303. doi: 10.3389/fpsyt.2022.913303, PMID: 35836656 PMC9275364

[ref163] Myrga J. M. Failla M. D. Ricker J. H. Dixon C. E. Yvette C. Patricia A.. (2016). A dopamine pathway gene risk score for cognitive recovery following traumatic brain injury: methodological considerations, preliminary findings, and interactions with sex. J. Head Trauma Rehabil. 31, E15–E29. doi: 10.1097/HTR.0000000000000199, PMID: 26580694

[ref164] Nair V. (1965). Regional changes in brain serotonin after head x-irradiation and its significance in the potentiation of barbiturate hypnosis. Nature 208, 1293–1294. doi: 10.1038/2081293a0 5870183

[ref165] Nakamura M. Yoshimi A. Tokura T. Kimura H. Kishi S. Tomoyad M.. (2024). Duloxetine improves chronic orofacial pain and comorbid depressive symptoms in association with reduction of serotonin transporter protein through upregulation of ubiquitinated serotonin transporter protein. Pain 165, 1177–1186. doi: 10.1097/j.pain.0000000000003124, PMID: 38227563

[ref166] Nyman E. S. Sulkava S. Soronen P. Miettunen J. Loukola A. Leppä V.. (2011). Interaction of early environment, gender and genes of monoamine neurotransmission in the aetiology of depression in a large population-based Finnish birth cohort. BMJ Open 1:e000087. doi: 10.1136/bmjopen-2011-000087, PMID: 22021758 PMC3191433

[ref167] Okada S. Morinobu S. Fuchikami M. Segawa M. Yokomaku K. Kataoka T.. (2014). The potential of SLC6A4 gene methylation analysis for the diagnosis and treatment of major depression. J. Psychiatr. Res. 53, 47–53. doi: 10.1016/j.jpsychires.2014.02.002, PMID: 24657235

[ref168] Pandya C. D. Hoda N. Crider A. Peter D. Kutiyanawalla A. Kumar S.. (2017). Transglutaminase 2 overexpression induces depressive-likebehavior and impaired TrkB signaling in mice. Mol. Psychiatry 22, 745–753. doi: 10.1038/mp.2016.145 27620841 PMC5348279

[ref169] Paresh D. P. Bochar D. A. Turner D. L. Meng F. Mueller H. M. Pontrello C. G. (2007). Regulation of tryptophan hydroxylase-2 gene expression by a bipartite RE-1 silencer of transcription/neuron restrictive silencing factor (REST/NRSF) binding motif. J. Biol. Chem. 282, 26717–26724. doi: 10.1074/jbc.M705120200, PMID: 17613521

[ref170] Pariante C. M. Lightman S. L. (2008). The HPA axis in major depression: classical theories and new developments. Trends Neurosci. 31, 464–468. doi: 10.1016/j.tins.2008.06.006, PMID: 18675469

[ref171] Park I. Choi M. Kim J. Jang S. Kim D. Kim J.. (2024). Role of the circadian nuclear receptor REV-ERBα in dorsal raphe serotonin synthesis in mood regulation. Commun. Biol. 7:998. doi: 10.1038/s42003-024-06647-y, PMID: 39147805 PMC11327353

[ref172] Park S. Kim Y. Lee J. Lee J. Y. Kim H. Lee S.. (2021). A systems biology approach to investigating the interaction between serotonin synthesis by tryptophan hydroxylase and the metabolic homeostasi. Int. J. Mol. Sci. 22:2452. doi: 10.3390/ijms22052452, PMID: 33671067 PMC7957782

[ref173] Park H. R. Lee H. Cho W. K. Ma J. Y. (2023). Pro-neurogenic effects of Lilii Bulbus on hippocampal neurogenesis and memory. Biomed. Pharmacother. 164:114951. doi: 10.1016/j.biopha.2023.114951 37267636

[ref174] Passarelli F. Angeletti B. Orrù D. Orzi F. D'Ambrosio E. (1994). Effects of electroconvulsive shock on the levels of hsp70 and hsc73 mRNA in the rat brain. Neurosci. Lett. 177, 147–150. doi: 10.1016/0304-3940(94)90888-5, PMID: 7824168

[ref175] Patel R. S. Krause-Hauch M. Kenney K. Miles S. Richardson R. N. Patel N. A. (2024). Long noncoding RNA VLDLR-AS1 levels in serum correlate with combat-related chronic mild traumatic brain injury and depression symptoms in US veterans. Int. J. Mol. Sci. 25:1473. doi: 10.3390/ijms25031473, PMID: 38338752 PMC10855201

[ref176] Pereira P. D. A. Silva M. A. R. Bicalho M. A. Marco L. D. Correa H. Campos S. B. D.. (2011). Association between tryptophan hydroxylase-2 gene and late-onset depression. Am. J. Geriatr. Psychiatry 19, 825–829. doi: 10.1097/JGP.0b013e31820eeb21, PMID: 21873838

[ref177] Peroutka S. J. Snyder S. H. (1979). Multiple serotonin receptors: differential binding of [^3^H]5-hydroxytryptamine, [^3^H]lysergic acid diethylamide and [^3^H]spiroperidol. Mol. Pharmacol. 16, 687–699. doi: 10.1016/S0026-895X(25)13812-1, PMID: 530254

[ref178] Peter D. Liu Y. Sternini C. Giorgio R. D. Brecha N. Edwards R. H. (1995). Differential expression of two vesicular monoamine transporters. J. Neurosci. 15, 6179–6188. doi: 10.1523/JNEUROSCI.15-09-06179.1995, PMID: 7666200 PMC6577657

[ref179] Ping L. L. Xu J. Zhou C. Lu J. Lu Y. Shen Z.. (2019). Tryptophan hydroxylase-2 polymorphism is associated with white matter integrity in first-episode, medication-naïve major depressive disorder patients. Psychiatry Res. Neuroimaging 286, 4–10. doi: 10.1016/j.pscychresns.2019.02.002, PMID: 30822678

[ref180] Poeggele B. Kumar S. Pappolla M. A. (2022). Tryptophan in nutrition and health. Int. J. Mol. Sci. 23:5455. doi: 10.3390/ijms23105455, PMID: 35628285 PMC9146092

[ref181] Portugalov A. Zaidan H. Gaisler-Salomon I. Hillard C. J. Akirav I. (2022). FAAH inhibition restores early life stress-induced alterations in PFC microRNAs associated with depressive-like behavior in male and female rats. Int. J. Mol. Sci. 23:6101. doi: 10.3390/ijms232416101, PMID: 36555739 PMC9782513

[ref182] Quinlan M. A. Robson M. J. Ye R. Rose K. L. Schey K. L. Blakely R. D. (2020). *Ex vivo* quantitative proteomic analysis of serotonin transporter Interactome: network impact of the SERT Ala56 coding variant. Front. Mol. Neurosci. 13:89. doi: 10.3389/fnmol.2020.00089, PMID: 32581705 PMC7295033

[ref183] Rajamanickam J. Annamalai B. Clemmensen T. R. Sundaramurthy S. Gether U. Jayanthi L. D.. (2015). Akt-mediated regulation of antidepressant-sensitive serotonin transporter function, cell-surface expression and phosphorylation. Biochem. J. 468, 177–190. doi: 10.1042/BJ20140826, PMID: 25761794 PMC13221098

[ref184] Ramamoorthy S. Shippenberg T. S. Jayanthi L. D. (2011). Regulation of monoamine transporters: role of transporter phosphorylation. Pharmacol. Ther. 129, 220–238. doi: 10.1016/j.pharmthera.2010.09.009, PMID: 20951731 PMC3031138

[ref185] Rampersaud R. Suneson K. Wu G. W. Y. Reus V. I. Lindqvist D. Ho T. C.. (2025) Kynurenine metabolism is associated with antidepressant response to selective serotonin reuptake inhibitors. *bioRxiv*. Available online at: 10.1101/2025.01.11.632543. [Epub ahead of preprint]

[ref186] Randesi M. Brink W. V. D. Levran O. Blanken P. Ree J. M. V. Ott J. (2019). VMAT2 gene (SLC18A2) variants associated with a greater risk for developing opioid dependence. Pharmacogenomics 20, 331–341. doi: 10.2217/pgs-2018-0137, PMID: 30983500 PMC6566135

[ref187] Rapport M. M. Green A. A. PAGE I. H. (1948). Crystalline serotonin. Science 108, 329–330. doi: 10.1126/science.108.2804.329, PMID: 17748034

[ref188] Reisinger S. N. Kong E. Molz B. Humberg T. Sideromenos S. Cicvaric A.. (2019). Flotillin-1 interacts with the serotonin transporter and modulates chronic corticosterone response. Genes Brain Behav. 18:e12482. doi: 10.1111/gbb.12482, PMID: 29667320 PMC6392109

[ref189] Ribases M. Ramos-Quiroga J. A. Hervas A. Bosch R. Bielsa A. Gastaminza X.. (2009). Exploration of 19 serotoninergic candidate genes in adults and children with attention-deficit/hyperactivity disorder identifies association for 5HT2A, DDC and MAOB. Mol. Psychiatry 14, 71–85. doi: 10.1038/sj.mp.4002100, PMID: 17938636

[ref190] Rizzi S. Spagnoli C. Frattini D. Pisani F. Fusco C. (2022). Clinical features in aromatic L-amino acid decarboxylase (AADC) deficiency: a systematic review. Behav. Neurol. 2022:2210555. doi: 10.1155/2022/2210555 36268467 PMC9578880

[ref191] Robson M. J. Quinlan M. A. Margolis K. G. Blakely R. D. (2018). p38α MAPK signaling drives pharmacologically reversible brain and gastrointestinal phenotypes in the SERT Ala56 mouse. Proc. Natl. Acad. Sci. U.S.A. 115, E10245–E10254. doi: 10.1073/pnas.1809137115, PMID: 30297392 PMC6205438

[ref192] Rodrigues P. Guimarães L. Carvalho A. P. Teles L. O. (2023). Carbamazepine, venlafaxine, tramadol, and their main metabolites: toxicological effects on zebrafish embryos and larvae. J. Hazard. Mater. 448:130909. doi: 10.1016/j.jhazmat.2023.130909, PMID: 36860067

[ref193] Roubertie A. Anselm I. Zeev B. B. Hwu W. L. Kumar A. Monteleone B.. (2024). Patient selection considerations for AADC deficiency gene therapy. Ann. Child Neurol. Soc. 2, 53–59. doi: 10.1002/cns3.20052, PMID: 40183958

[ref194] Roumier A. Béchade C. Maroteaux L. (2019). Serotonin and the immune system, in Serotonin, ed. Pilowsky P. M. (San Diego, CA: Academic Press), 181–196.

[ref195] Royal College of Psychiatrists (2019). Position statement on antidepressants and depression. London: Royal College of Psychiatrists.

[ref196] Rucci P. Nimgaonkar V. L. Mansour H. Miniati M. Masala I. Fagiolini A.. (2009). Gender moderates the relationship between mania spectrum and serotonin transporter polymorphisms in depression. Am. J. Med. Genet. B 150B, 907–913. doi: 10.1002/ajmg.b.30917, PMID: 19125390 PMC3387576

[ref197] Ryan K. M. Corrigan M. Murphy T. M. McLoughlin D. M. Harkin A. (2024). Gene expression of kynurenine pathway enzymes in depression and following electroconvulsive therapy. Acta Neuropsychiatr. 37, 1–10. doi: 10.1017/neu.2024.34 PMC13130249 39417574

[ref198] Sager J. J. Torres G. E. (2011). Proteins interacting with monoamine transporters: current state and future challenges. Biochemistry 50, 7295–7310. doi: 10.1021/bi200405c, PMID: 21797260

[ref199] Saldanha D. Kumar N. Ryali V. Srivastava K. Pawar A. A. (2009). Serum serotonin abnormality in depression. Med. J. Armed Forces India 65, 108–112. doi: 10.1016/S0377-1237(09)80120-2, PMID: 27408213 PMC4921409

[ref200] Sanwald S. Müller K. W. Lecuona C. S. Montag C. Kiefer M. GenEmo Research Group (2021). Factors related to age at depression onset: the role of SLC6A4 methylation, sex, exposure to stressful life events and personality in a sample of inpatients suffering from major depression. BMC Psychiatry 21:220. doi: 10.1186/s12888-021-03215-0 33926413 PMC8086353

[ref201] Saroj N. Shanker S. Hernández E. S. Gutiérrez G. M. Mondragón J. A. Martínez S. M.. (2025). Expression of tryptophan hydroxylase in rat adrenal glands: upregulation of TPH2 by chronic stress. Psychoneuroendocrinology 171:107219. doi: 10.1016/j.psyneuen.2024.107219 39467477

[ref202] Schaefer T. L. Vorhees C. V. Williams M. T. (2009). Mouse Pet-1 knock-out induced 5-HT disruption results in a lack of cognitive deficits and an anxiety phenotype complicated by hypoactivity and defensiveness. Neuroscience 164, 1431–1443. doi: 10.1016/j.neuroscience.2009.09.059, PMID: 19786075 PMC2783314

[ref203] Schäfer M. K. H. Hartwig N. R. Kalmbach N. Klietz M. Anlauf M. Eiden L. E.. (2014). Species-specific vesicular monoamine transporter 2 (VMAT2) expression in mammalian pancreatic beta cells: implications for optimising radioligand-based human beta cell mass (BCM) imaging in animal models. Diabetologia 56, 1047–1056. doi: 10.1007/s00125-013-2847-7 PMC3955760 23404442

[ref204] Schildkraut J. J. (1965). The catecholamine hypothesis of affective disorders: A review of supporting evidence. Am. J. Psychiatry 122, 509–522. doi: 10.1176/ajp.122.5.509, PMID: 5319766

[ref205] Schuch J. B. Müller D. Endres R. G. Bosa C. A. (2016). Psychomotor agitation and mood instability in patients with autism spectrum disorders: a possible effect of SLC6A4 gene? Res. Autism Spectr. Disord. 26, 48–56. doi: 10.1016/j.rasd.2016.03.001

[ref206] Schultz J. L. Killoran A. Nopoulos P. C. Chabal C. C. Moser D. J. Kamholz J. A. (2018). Evaluating depression and suicidality in tetrabenazine users with Huntington disease. Neurology 91, e202–e207. doi: 10.1212/WNL.0000000000005817, PMID: 29925548

[ref207] Schwab S. G. Franke P. E. Hoefgen B. Guttenthaler V. Lichtermann D. Trixler M.. (2005). Association of DNA polymorphisms in the synaptic vesicular amine transporter gene (SLC18A2) with alcohol and nicotine dependence. Neuropsychopharmacology 30, 2263–2268. doi: 10.1038/sj.npp.1300809, PMID: 15988470

[ref208] Schwartz K. Yadid G. Weizman A. Rehavi M. (2003). Decreased limbic vesicular monoamine transporter 2 in a genetic rat model of depression. Brain Res. 965, 174–179. doi: 10.1016/S0006-8993(02)04167-7, PMID: 12591135

[ref209] Shaif N. A. Jang D. Cho D. Kim S. Seo D. B. Shim I. (2018). The antidepressant-like effect of lactate in an animal model of menopausal depression. Biomedicines 6:21108. doi: 10.3390/biomedicines6040108, PMID: 30469388 PMC6316721

[ref210] Sharp T. Barnes N. M. (2020). Central 5-HT receptors and their function; present and future. Neuropharmacology 177:108155. doi: 10.1016/j.neuropharm.2020.108155, PMID: 32522572

[ref211] Shaw C. Hess M. Weimer B. C. (2023). Microbial-derived tryptophan metabolites and their role in neurological disease: anthranilic acid and anthranilic acid derivatives. Microorganisms 11:1825. doi: 10.3390/microorganisms11071825, PMID: 37512997 PMC10384668

[ref212] Shen J. Zhang B. Chen J. Cheng J. Wang J. Zheng X.. (2022). SAHA alleviates diarrhea-predominant irritable bowel syndrome through regulation of the p-STAT3/SERT/5-HT signaling pathway. J. Inflamm. Res. 15, 1745–1756. doi: 10.2147/JIR.S331303, PMID: 35300211 PMC8923685

[ref213] Sheng Y. Yvette P. Paul S. M. Cooper B. A. Carpenter J. S. Hammer M. J.. (2024). Palpitations in women with breast cancer are associated with polymorphisms for neurotransmitter genes. Oncol. Nurs. Forum 51, 332–348. doi: 10.1188/24.ONF.332-348, PMID: 38950091

[ref214] Shin M. S. Park S. S. Lee J. M. Kim T. W. Kim Y. P. (2017). Treadmill exercise improves depression-like symptoms by enhancing serotonergic function through upregulation of 5-HT1A expression in the olfactory bulbectomized rats. J. Exerc. Rehabil. 13, 36–42. doi: 10.12965/jer.1734918.459, PMID: 28349031 PMC5331997

[ref215] Siemann J. K. William P. Malik T. N. Jackson C. R. Green N. H. Emeson R. B.. (2020). Photoperiodic effects on monoamine signaling and gene expression throughout development in the serotonin and dopamine systems. Sci. Rep. 10:15437. doi: 10.1038/s41598-020-72263-5, PMID: 32963273 PMC7508939

[ref216] Singh S. B. Tiwari A. Katta M. R. Kafle R. Ayubcha C. Patel K. H.. (2024). The utility of PET imaging in depression. Front. Psychiatry 15:1322118. doi: 10.3389/fpsyt.2024.1322118, PMID: 38711875 PMC11070570

[ref217] Soleimani L. Roder J. C. Dennis J. W. Lipina T. (2008). Beta N-acetylglucosaminyltransferase V (Mgat5) deficiency reduces the depression-like phenotype in mice. Genes Brain Behav. 7, 334–343. doi: 10.1111/j.1601-183X.2007.00358.x, PMID: 17883406

[ref218] Song M. F. Dong J. Z. Wang Y. W. He J. Ju X. Zhang L.. (2015). CSF miR-16 is decreased in major depression patients and its neutralization in rats induces depression-like behaviors via a serotonin transmitter system. J. Affect. Disord. 178, 25–31. doi: 10.1016/j.jad.2015.02.022, PMID: 25779937

[ref219] Song N. N. Huang J. B. Y. Chen J. Y. Zhang L. Gutknecht L. Lesch K. P.. (2014). Adult raphe-specific deletion of Lmx1b leads to central serotonin deficiency. PLoS One 9:e104318. doi: 10.1371/journal.pone.0015998 21246047 PMC3016403

[ref220] Specker E. Wesolowski R. Schütz A. Matthes S. Mallow K. Wasinska K. M.. (2023). Structure-based design of xanthine-imidazopyridines and-imidazothiazoles as highly potent and *in vivo* efficacious tryptophan hydroxylase inhibitors. J. Med. Chem. 66, 14866–14896. doi: 10.1021/acs.jmedchem.3c01454, PMID: 37905925

[ref221] Stefanovic B. Spasojevic N. Jovanovic P. Jasnic N. Djordjevic J. Dronjak S. (2016). Melatonin mediated antidepressant-like effect in the hippocampus of chronic stress-induced depression rats: regulating vesicular monoamine transporter 2 and monoamine oxidase A levels. Eur. Neuropsychopharmacol. 26, 1629–1637. doi: 10.1016/j.euroneuro.2016.07.005, PMID: 27499503

[ref222] Steinbusch H. W. M. Dolatkhah M. A. Hopkins D. A. (2021). Anatomical and neurochemical organization of the serotonergic system in the mammalian brain and in particular the involvement of the dorsal raphe nucleus in relation to neurological diseases. Prog. Brain Res. 261, 41–81. doi: 10.1016/bs.pbr.2021.02.003, PMID: 33785137

[ref223] Stockmeier C. A. (1997). Neurobiology of serotonin in depression and suicide. Ann. N. Y. Acad. Sci. 836, 220–232. doi: 10.1111/j.1749-6632.1997.tb52362.x, PMID: 9616801

[ref224] Su S. Zhao J. Bremner J. D. Miller A. H. Tang W. Bouzyk M.. (2009). Serotonin transporter gene, depressive symptoms, and interleukin-6. Circ. Cardiovasc. Genet. 2, 614–620. doi: 10.1161/CIRCGENETICS.109.870386, PMID: 20031642 PMC2802220

[ref225] Suga H. Asakura K. Kobayashi S. Nojima M. Sasaki S. (2018). Association between habitual tryptophan intake and depressive symptoms in young and middle-aged women. J. Affect. Disord. 231, 44–50. doi: 10.1016/j.jad.2018.01.029, PMID: 29438897

[ref226] Suganya S. Ashok B. S. Ajith T. A. (2024). A recent update on the role of Estrogen and progesterone in Alzheimer’s disease. Cell Biochem. Funct. 42:e70025. doi: 10.1002/cbf.70025, PMID: 39663597

[ref227] Sun N. Qin Y. A. J. Xu C. Xia T. Du Z. W. Zheng L. P.. (2022). Design of fast-onset antidepressant by dissociating SERT from nNOS in the DRN. Science 378, 390–398. doi: 10.1126/science.abo3566, PMID: 36302033

[ref228] Tahiri J. Mian M. Aftan F. Habbal S. Salehi F. Reddy P. H.. (2024). Serotonin in depression and Alzheimer's disease: focus on SSRI’s beneficial effects. Ageing Res. Rev. 101:102537. doi: 10.1016/j.arr.2024.102537 39389238 PMC11531385

[ref229] Talkowski M. E. Kirov G. Bamne M. Georgieva L. Torres G. Mansour H.. (2008). A network of dopaminergic gene variations implicated as risk factors for schizophrenia. Hum. Mol. Genet. 17, 747–758. doi: 10.1093/hmg/ddm347, PMID: 18045777 PMC3777405

[ref230] Tanaka M. Bohár Z. Vécsei L. (2020). Are kynurenines accomplices or principal villains in dementia? Maintenance of kynurenine metabolism. Molecules 25:564. doi: 10.3390/molecules25030564, PMID: 32012948 PMC7036975

[ref231] Tang Y. Q. Li Z. R. Zhang S. Z. Mi P. Chen D. Y. Feng X. Z. (2019). Venlafaxine plus melatonin ameliorate reserpine-induced depression-like behavior in zebrafish. Neurotoxicol. Teratol. 76:106835. doi: 10.1016/j.ntt.2019.106835, PMID: 31518687

[ref232] Tao L. Yao C. Wang S. Ye Y. Tu Z. Jiang X.. (2024). Synthesis and biological evaluation of novel isobenzofuran-1(3H)-one derivatives as antidepressant agents. Bioorg. Med. Chem. 114:117941. doi: 10.1016/j.bmc.2024.117941, PMID: 39432939

[ref233] Tikker L. Casarotto P. Singh P. Biojone C. Piepponen T. P. Estartús N.. (2020). Inactivation of the GATA cofactor ZFPM1 results in abnormal development of dorsal raphe serotonergic neuron subtypes and increased anxiety-like behavior. J. Neurosci. 40, 8669–8682. doi: 10.1523/JNEUROSCI.2252-19.2020, PMID: 33046550 PMC7643297

[ref234] Toma C. Hervás A. Balmaña N. Salgado M. Maristany M. Vilella E.. (2013). Neurotransmitter systems and neurotrophic factors in autism: association study of 37 genes suggests involvement of DDC. World J. Biol. Psychiatry 14, 516–527. doi: 10.3109/15622975.2011.602719, PMID: 22397633

[ref235] Uliana D. L. Martinez A. Grace A. A. (2024). THPP-1 PDE10A inhibitor reverses the cognitive deficits and hyperdopaminergic state in a neurodevelopment model of schizophrenia. Schizophr. Res. 274, 315–326. doi: 10.1016/j.schres.2024.10.003, PMID: 39437478 PMC12444882

[ref236] Velazquez D. C. Becerra J. P. (2024). Paradoxical boosting of weak and strong spatial memories by hippocampal dopamine uncaging. eNeuro 11, ENEURO.0469–ENEU23.2024. doi: 10.1523/ENEURO.0469-23.2024, PMID: 38755011 PMC11138129

[ref237] Vialli M. Erspamer V. (1937). Ricerche sul secreto delle cellule enterocromaffini: Nota VII Osservazioni critiche su alcuni problemi inerenti alla istochimica delle enterocromaffini. Z. Für Zellforsch. Mikrosk. 27, 81–99.

[ref238] Vijayan N. N. Iwayama Y. Koshy L. V. Natarajan C. Nair C. Allencherry P. M.. (2009). Evidence of association of serotonin transporter gene polymorphisms with schizophrenia in a south Indian population. J. Hum. Genet. 54, 538–542. doi: 10.1038/jhg.2009.76, PMID: 19713975

[ref239] Wabel E. A. Burke T. K. Watts S. W. (2024). Vascular chemerin from PVAT contributes to norepinephrine and serotonin-induced vasoconstriction and vascular stiffness in a sex-dependent manner. Am. J. Physiol. Heart Circ. Physiol. 327, H1577–H1589. doi: 10.1152/ajpheart.00475.2024, PMID: 39453435

[ref240] Walthe D. J. Peter J. U. Bashammakh S. Hörtnagl H. Voits M. Fink H.. (2003). Synthesis of serotonin by a second tryptophan hydroxylase isoform. Science 299:76. doi: 10.1126/science.1078197 12511643

[ref241] Wandowski A. M. Zander J. F. Richter K. Hilger G. A. (2016). Co-existence of functionally different vesicular neurotransmitter transporters. Front. Synaptic Neurosci. 8:4. doi: 10.3389/fnsyn.2016.00004 26909036 PMC4754932

[ref242] Wang T. M. Gainetdinov R. R. Fumagalli F. Xu F. Jones S. R. Bock C. B.. (1997). Knockout of the vesicular monoamine transporter 2 gene results in neonatal death and super sensitivity to cocaine and amphetamine. Neuron 19, 1285–1296. doi: 10.1016/S0896-6273(00)80419-5, PMID: 9427251

[ref243] Wang Y. Li S. Liu W. Wang F. Hu L. F. Zhong Z.. (2016). Vesicular monoamine transporter 2 (Vmat2) knockdown elicits anxiety-like behavior in zebrafish. Biochem. Biophys. Res. Commun. 470, 792–797. doi: 10.1016/j.bbrc.2016.01.079, PMID: 26801555

[ref244] Wang M. Sun Y. Hu B. He Z. Chen S. Qi D.. (2022). Organic cation transporters are involved in fluoxetine transport across the blood-brain barrier *in vivo* and *in vitro*. Curr. Drug Deliv. 19, 508–517. doi: 10.2174/1567201818666210708122326, PMID: 34238184

[ref245] Wang Q. Sun Y. N. Zou C. M. Zhang T. L. Li Z. Liu M.. (2022). Regulation of the kynurenine/serotonin pathway by berberine and the underlying effect in the hippocampus of the chronic unpredictable mild stress mice. Behav. Brain Res. 422:113764. doi: 10.1016/j.bbr.2022.113764, PMID: 35051489

[ref246] Wang Y. Wang D. Zhang X. Li H. Wang S. He Y.. (2024). Dorsal raphe serotonergic neurons-ventral tegmental area neural pathway promotes wake from sleep. CNS Neurosci. Ther. 30:e70141. doi: 10.1111/cns.70141, PMID: 39593192 PMC11598740

[ref247] Wang L. Zhang M. Zhu H. Sun L. Yu B. Cui X. (2021). Combined identification of lncRNA NONHSAG004550 and NONHSAT125420 as a potential diagnostic biomarker of perinatal depression. J. Clin. Lab. Anal. 35:e23890. doi: 10.1002/jcla.23890, PMID: 34263944 PMC8373316

[ref248] Wei J. Chu C. Wang Y. Yang Y. Wang Q. Li T.. (2012). Association study of 45 candidate genes in nicotine dependence in Han Chinese. Addict. Behav. 37, 622–626. doi: 10.1016/j.addbeh.2012.01.009, PMID: 22309839

[ref249] Wichers M. C. Koek G. H. Robaeys G. Verkerk R. Scharpé S. Maes M. (2005). IDO and interferon-α-induced depressive symptoms: a shift in hypothesis from tryptophan depletion to neurotoxicity. Mol. Psychiatry 10, 538–544. doi: 10.1038/sj.mp.4001600, PMID: 15494706

[ref250] Wigner P. Czarny P. Synowiec E. Bijak M. Białek K. Talarowska M.. (2018). Association between single nucleotide polymorphisms of TPH1and TPH2 genes, and depressive disorders. J. Cell. Mol. Med. 22, 1778–1791. doi: 10.1111/jcmm.13459, PMID: 29314569 PMC5824396

[ref251] Witt C. E. Mena S. Holmes J. Hersey M. Buchanan A. M. Parke B.. (2023). Serotonin is a common thread linking different classes of antidepressants, cell. Chem. Biol. 30, 1557–1570.e6. doi: 10.1016/j.chembiol.2023.10.009, PMID: 37992715

[ref252] Won E. Han K. M. Kang J. Kim A. Yoon H. K. Chang H. S.. (2017). Vesicular monoamine transporter 1 gene polymorphism and white matter integrity in major depressive disorder. Prog. Neuropsychopharmacol. Biol. Psychiatry 77, 138–145. doi: 10.1016/j.pnpbp.2017.02.028, PMID: 28408293

[ref253] Wong D. T. Bymaster F. P. Engleman E. A. (1995). Prozac (fluoxetine, Lilly 110140), the first selective serotonin uptake inhibitor and an antidepressant drug: twenty years since its first publication. Life Sci. 57, 411–441. doi: 10.1016/0024-3205(95)00209-o, PMID: 7623609

[ref254] Wood J. D. (2007). Enteric nervous system, serotonin, and the irritable bowel syndrome. Curr. Opin. Gastroenterol. 23, 121–126. doi: 10.1097/MOG.0b013e3280287a23, PMID: 17031157

[ref255] Wray N. R. James M. R. Gordon S. D. Dumenil T. Ryan L. Coventry W. L.. (2009). Accurate large-scale genotyping of 5HTTLPR and flanking single nucleotide polymorphisms in an association study of depression, anxiety, and personality measures. Biol. Psychiatry 66, 468–476. doi: 10.1016/j.biopsych.2009.04.030, PMID: 19541292 PMC3060567

[ref256] Wróbel M. Z. Chodkowski A. Siwek A. Satała G. Bojarski A. J. Dawidowski M. (2024). Design and synthesis of potential multi-target antidepressants: exploration of 1-(4-(7-azaindole)-3,6-dihydropyridin-1-yl)alkyl-3-(1H-indol-3-yl)pyrrolidine-2,5-dione derivatives with affinity for the serotonin transporter. Int. J. Mol. Sci. 25:11276. doi: 10.3390/ijms252011276, PMID: 39457057 PMC11508649

[ref257] Xia T. J. Jin S. W. Liu Y. G. Zhang S. S. Wang Z. Liu X. M.. (2024). Shen Yuan extract exerts a hypnotic effect via the tryptophan/5-hydroxytryptamine/melatonin pathway in mice. J. Ethnopharmacol. 326:117992. doi: 10.1016/j.jep.2024.117992, PMID: 38428654

[ref258] Xia S. Maitiniyazi G. Liu Y. Chen Y. Guo M. He J.. (2023). Whey protein isolate attenuates depression-like behavior developed in a mouse model of breast tumor. Food Res. Int. 169:112849. doi: 10.1016/j.foodres.2023.112849 37254425

[ref259] Xu B. Gottschalk W. Chow A. Wilson R. I. Schnell E. Zang K.. (2000). The role of brain-derived neurotrophic factor receptors in the mature hippocampus: modulation of long-term potentiation through a presynaptic mechanism involving TrkB. J. Neurosci. 20, 6888–6897. doi: 10.1523/JNEUROSCI.20-18-06888.2000, PMID: 10995833 PMC2711895

[ref260] Xu Y. Han X. Zhu Y. Deng B. Li L. Du Y.. (2025). Chaihu Shugan San exerts antidepressant effects by regulating glucocorticoid metabolism in CUMS rats and network pharmacology provides complementary mechanistic insights. ACS Omega 10, 6780–6793. doi: 10.1021/acsomega.4c08802, PMID: 40028110 PMC11866020

[ref261] Yamamoto M. MSuhara T. Okub Y. Ichimiya T. Sudo Y. Inoue M.. (2002). Age-related decline of serotonin transporters in living human brain of healthy males. Life Sci. 71, 751–757. doi: 10.1016/S0024-3205(02)01745-9, PMID: 12074934

[ref262] Yi L. T. Li J. M. Li Y. C. Pan Y. Xu Q. Kong L. D. (2008). Antidepressant-like behavioral and neurochemical effects of the citrus-associated chemical apigenin. Life Sci 82, 741–751. doi: 10.1016/j.lfs.2008.01.007, PMID: 18308340

[ref263] Yohn S. E. Gorka D. Mistry A. Collins S. Qian E. Correa M.. (2017). Oral ingestion and intraventricular injection of curcumin attenuates the effort-related effects of the VMAT-2 inhibitor tetrabenazine: implications for motivational symptoms of depression. J. Nat. Prod. 80, 2839–2844. doi: 10.1021/acs.jnatprod.7b00425 28905625

[ref264] Yohn S. E. Thompson C. Randall P. A. Lee C. A. Müller C. E. Baqi Y.. (2015). The VMAT-2 inhibitor tetrabenazine alters effort-related decision making as measured by the T-maze barrier choice task: reversal with the adenosine A2A antagonist MSX-3 and the catecholamine uptake blocker bupropion. Psychopharmacology 232, 1313–1323. doi: 10.1007/s00213-014-3766-0 25323625

[ref265] Yu Y. Panhuysen C. Kranzler H. R. Hesselbrock V. Rounsaville B. Weiss R.. (2006). Intronic variants in the dopa decarboxylase (DDC)gene are associated with smoking behavior in European-Americans and African-Americans. Hum. Mol. Genet. 15, 2192–2199. doi: 10.1093/hmg/ddl144, PMID: 16740595

[ref266] Yun J. Y. Kim Y. K. (2024). Electroconvulsive therapy (ECT) in major depression: oldies but goodies. Adv. Exp. Med. Biol. 1456, 187–196. doi: 10.1007/978-981-97-4402-2_10, PMID: 39261430

[ref267] Zai C. C. Tiwari A. K. Mazzoco M. Luca V. D. Müller D. J. Shaikh S. A.. (2013). Association study of the vesicular monoamine transporter gene SLC18A2 with tardive dyskinesia. J. Psychiatr. Res. 47, 1760–1765. doi: 10.1016/j.jpsychires.2013.07.025, PMID: 24018103

[ref268] Zhan Z. Ye M. Jin X. (2023). The roles of FLOT1 in human diseases (Review). Mol. Med. Rep. 28:212. doi: 10.3892/mmr.2023.13099, PMID: 37772385 PMC10552069

[ref269] Zhang Y. Chen Y. Chen G. Zhou Y. Yao H. Tan H. (2020a). Upregulation of miR-361-3p suppresses serotonin-induced proliferation in human pulmonary artery smooth muscle cells by targeting SERT. Cell. Mol. Biol. Lett. 25:45. doi: 10.1186/s11658-020-00237-6, PMID: 33061998 PMC7542879

[ref270] Zhang X. Gainetdinov R. R. Beaulieu J. M. Sotnikova T. D. Burch L. H. Williams R. B. (2005). Loss-of-function mutation in tryptophan hydroxylase-2 identified in unipolar major depression. Neuron 45, 11–16. doi: 10.1016/j.neuron.2004.12.014, PMID: 15629698

[ref271] Zhang Z. W. Gao C. S. Zhang H. Yang J. Wang Y. P. Pan L. B.. (2022). *Morinda officinalis* oligosaccharides increase serotonin in the brain and ameliorate depression via promoting 5-hydroxytryptophan production in the gut microbiota. Acta Pharm. Sin. B 12, 3298–3312. doi: 10.1016/j.apsb.2022.02.032, PMID: 35967282 PMC9366226

[ref272] Zhang X. Wang M. Qiao Y. Shan Z. Yang M. Li G.. (2022). Exploring the mechanisms of action of *Cordyceps sinensis* for the treatment of depression using network pharmacology and molecular docking. Ann. Transl. Med. 10:282. doi: 10.21037/atm-22-762 35434037 PMC9011256

[ref273] Zhang Y. Zhang C. Yuan G. Yao J. Cheng Z. Liu C.. (2010). Effect of tryptophan hydroxylase-2 rs7305115 SNP on suicide attempts risk in major depression. Behav. Brain Funct. 6:49. doi: 10.1186/1744-9081-6-49, PMID: 20738857 PMC2939585

[ref274] Zhang Y. Zhang H. Zhang W. Zhang Y. Wang W. Nie L. (2020b). LncRNA XIST modulates 5-hydroxytrytophan-induced visceral hypersensitivity by epigenetic silencing of the SERT gene in mice with diarrhea-predominant IBS. Cell. Signal. 73:109674. doi: 10.1016/j.cellsig.2020.109674 32446903

[ref275] Zhanga Q. Sun Y. He Z. Xu Y. Li X. Ding J.. (2020). Kynurenine regulates NLRP2 inflammasome in astrocytes and its implications in depression. Brain Behav. Immun. 88, 471–481. doi: 10.1016/j.bbi.2020.04.016, PMID: 32283293

[ref276] Zhao J. Sun Y. Feng Y. Rong J. (2024). Brain specific RagA overexpression triggers depressive-like behaviors in mice via activating ADORA2A signaling pathway. Adv. Sci. 11:e2404188. doi: 10.1002/advs.202404188, PMID: 39373701 PMC11615787

[ref277] Zhao R. Zhou Y. Shi H H. Ye W. Lyu Y. Wen Z.. (2022). Effect of gestational diabetes on postpartum depression-like behavior in rats and its mechanism. Nutrients 14:1229. doi: 10.3390/nu14061229, PMID: 35334886 PMC8953401

[ref278] Zheng J. Y. Li X. X. Liu X. Zhang C. C. Sun Y. X. Ma Y. N.. (2024). Fluoxetine reverses early-life stress-induced depressive-like behaviors and region-specific alterations of monoamine transporters in female mice. Pharmacol. Biochem. Behav. 237:173722. doi: 10.1016/j.pbb.2024.173722, PMID: 38336220

[ref279] Zhong X. Cao W. Zhao H. Chen L. Cao J. Wei L.. (2020). MicroRNA-32-5p knockout eliminates lipopolysaccharide-induced depressive-like behavior in mice through inhibition of astrocyte overactivity. Brain Behav. Immun. 84, 10–22. doi: 10.1016/j.bbi.2019.11.001 31698013

[ref280] Zhong H. Sánchez C. Caron M. G. (2012). Consideration of allosterism and interacting proteins in the physiological functions of the serotonin transporter. Biochem. Pharmacol. 83, 435–442. doi: 10.1016/j.bcp.2011.09.020, PMID: 21983034

[ref281] Zou Z. Huang Y. Wang J. Min W. Zhou B. (2020). The association between serotonin-related gene polymorphisms and susceptibility and early sertraline response in patients with panic disorder. BMC Psychiatry 20:388. doi: 10.1186/s12888-020-02790-y, PMID: 32723321 PMC7388522

[ref282] Zou X. H. Sun L. H. Yang W. Li B. J. Cui R. J. (2020). Potential role of insulin on the pathogenesis of depression. Cell Prolif. 53:e12806. doi: 10.1111/cpr.12806, PMID: 32281722 PMC7260070

[ref283] Zurawek D. Kusmider M. Faron-Gorecka A. Gruca P. Pabian P. Solich J.. (2017). Reciprocal microRNA expression in mesocortical circuit and its interplay with serotonin transporter define resilient rats in the chronic mild stress. Mol. Neurobiol. 54, 5741–5751. doi: 10.1007/s12035-016-0107-9 27660265 PMC5583278

